# Improving flood detection with large-scale dashboard camera data

**DOI:** 10.1038/s41467-026-75938-1

**Published:** 2026-07-29

**Authors:** Matt Franchi, Nikhil Garg, Wendy Ju, Emma Pierson

**Affiliations:** 1 Ann S. Bowers College of Computing and Information Science, Cornell Tech, New York, NY USA; 2 School of Operations Research and Information Engineering, Cornell Tech, New York, NY USA; 3 Department of Electrical Engineering and Computer Sciences, University of California - Berkeley, Berkeley, CA USA

**Keywords:** Natural hazards, Computational science

## Abstract

Flooding poses a significant and growing challenge globally, threatening infrastructure, livelihoods, and public safety. However, current methods for detecting floods are limited in their spatiotemporal granularity and inequitable in their coverage. Here, we propose BayFlood, a method for fine-grained urban flood detection that identifies flooded street scenes in large-scale dashboard camera datasets using a vision-language model. We leverage the ability of modern vision-language models to identify floods even without large labeled datasets, which are typically unavailable. We comprehensively validate our approach using 1,440,184 images, showing that our model provides strong signal for floods across multiple cities and time periods and that our flood detections correlate with known external predictors of flood risk. We show our approach can be used to improve flood detection in New York City: our analysis detects floods in neighborhoods overlooked by current methods, identifies demographic biases in existing methods, and suggests locations for new flood sensors. This work underscores the potential of leveraging dense street-level imagery to significantly improve the understanding, detection, and management of flooding, and is more broadly applicable to detecting other objects and incidents from street scene data even when no labeled data is available.

## Abstract (web summary)

This study shows that BayFlood, a fine-grained urban flood detection method that uses machine learning to identify flooded street scenes in large-scale dashboard camera datasets, can detect floods missed by conventional methods and help identify locations for new flood sensors.

## Introduction

Flooding is a significant and escalating global issue, endangering lives and causing serious economic impacts^1–4^. Since 2000, flooding has affected 1.6 billion people globally, caused at least $651 billion in damages, and led to more than 130,000 fatalities^5–7^. Within the United States alone, flooding costs more than $180 billion annually^8^ and causes more deaths than any other weather hazard except heat^9^. As climate change intensifies rainfall patterns, urban areas in particular are increasingly susceptible to frequent and severe flooding events, highlighting the urgency of effective monitoring and response strategies^1,3^.

But existing methods for detecting urban flooding are inadequate. Cities currently rely on several methods for detecting and responding to floods, each with its own limitations. One tool for flood detection is flood sensors: while these provide precise and continuous measurements at specific locations, they suffer from insufficient spatiotemporal granularity because it is not feasible to place sensors everywhere in a city. For example, there are roughly 40,000 intersections in New York City^10^, but the city’s FloodNet sensor network currently has only around 250 sensors deployed and is expanding to over 500 sensors citywide at a cost of $7.2 million^11^. A second source of flood information is crowd-sourced reporting systems, such as 311^12,13^, which allow city residents to report problems. These reporting systems offer higher spatiotemporal granularity, but are prone to biases: differential reporting rates across neighborhoods and demographic groups can lead to an inequitable and misleading representation of flood impacts^14–18^. A third source of information is high-resolution physics-based flood forecasting models^19–23^, including predicted stormwater accumulation zones. While the predictive capabilities of these models continue to improve, open challenges come from imperfect modeling of where rainfall will actually occur^24^ (e.g., the New York City Department of Environmental Protection (DEP)’s maps assume uniform rainfall over the entire city^23^); sewer network model simplifications that are necessary for efficient computation but undermine accuracy^25^; and omission of important but difficult-to-capture factors like clogged catch basins^14,24^. Collectively, these limitations of existing methods create an urgent need for new approaches to enhance flood detection and response capabilities, especially in the face of more intense weather patterns from climate change^26^.

A promising option for improving flood detection is using computer vision to identify flooded images in large-scale dashboard camera (dashcam) datasets, enabling fine-grained monitoring of flood conditions across urban areas. Dashcam data offer high spatiotemporal granularity, capturing street-level conditions continuously across broad urban areas^27^. A rich prior literature using street scene imagery to provide insight into urban life^28–43^ suggests the potential of this approach. However, using computer vision to detect floods from dashcam data requires overcoming a core technical challenge: the lack of large labeled datasets typically used to train computer vision models^44^. Even on days when flooding occurs, most dashcam images do not show flooding, making it difficult to curate datasets with enough flooded images for model training^45^; reliable ground-truth information on where floods occur is also lacking, due to the limitations of existing detection methods, making it difficult to obtain accurate labels.

Now, the zero-shot classification abilities of modern vision-language models (VLMs)^46–48^ suggest a way of overcoming this challenge. These models are pretrained on large corpora of images and text, enabling them to perform zero-shot classification and complex semantic reasoning without task-specific fine-tuning. Past work has investigated whether VLMs can characterize flooding using images from sources including surveillance cameras, community science initiatives, or Google Images^49–51^, yielding mixed results: VLMs have shown good performance on flood detection^49^ but less reliable performance on more challenging tasks like flood depth estimation^50,51^. This work is also limited by relatively small datasets, a lack of spatial granularity (e.g., due to sparse surveillance cameras), or non-representative images (e.g., from assorted Google Images), making it unclear whether VLMs are effective in more realistic flood-detection settings.

**Fig. 1:**
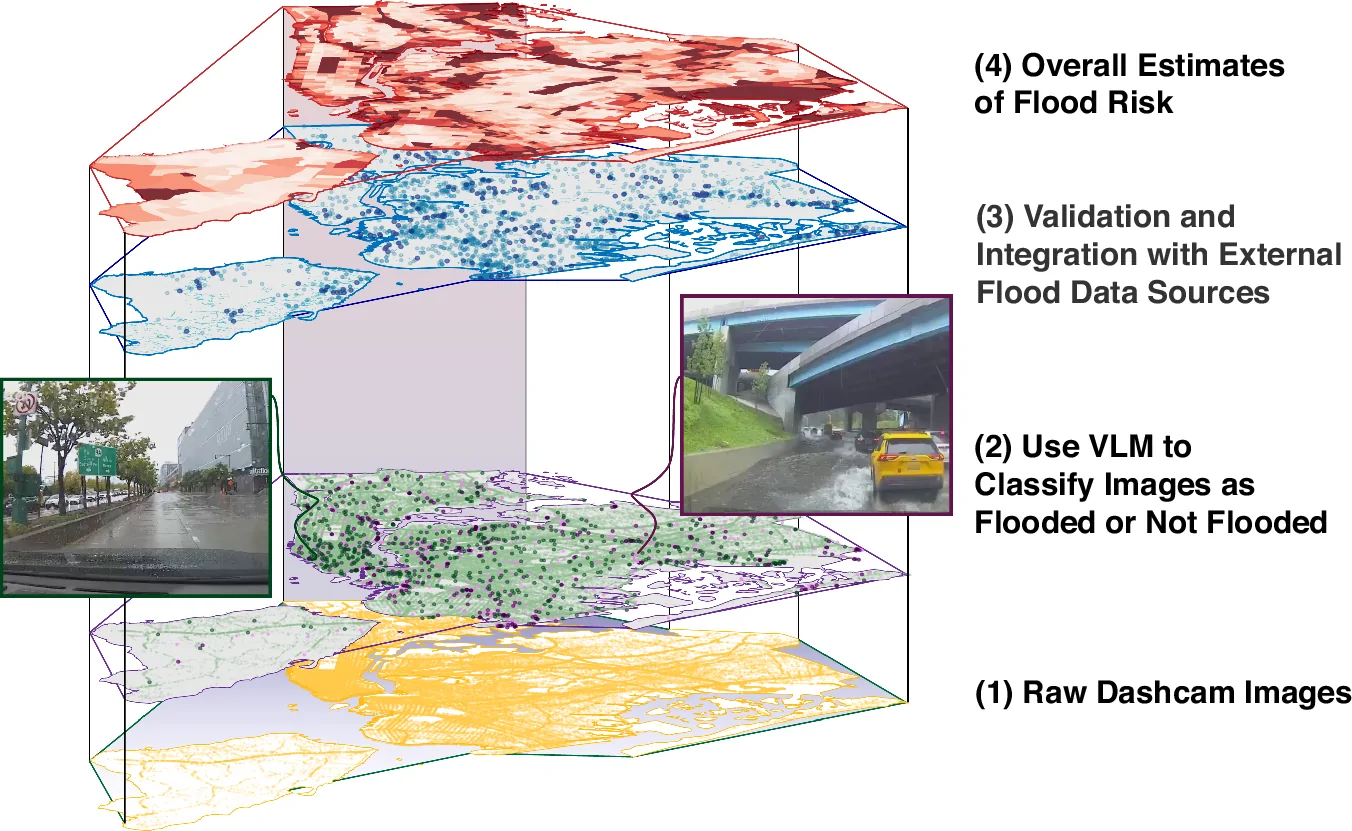
Overview of the flood detection pipeline. Dashcam images (layer 1, from bottom) are classified by a computer vision model (layer 2) as flooded (purple) or non-flooded (green). These image classifications are then validated against external data (layer 3) --- including ground-truth human annotations of the images, stormwater predictions, flood sensor locations, and resident complaints of flooding. All data sources are combined to produce the final estimates of flooded fraction within each Census tract (layer 4).

Here, we propose a flood-detection method, BayFlood, that performs well on realistic, large-scale dashcam data and yields improvements in practical urban flood-detection tasks. BayFlood pairs zero-shot VLM classification with a spatial Bayesian model that incorporates external flood-relevant data sources (Fig. 1). We comprehensively validate BayFlood using a uniquely large and spatiotemporally granular dataset of 1,440,184 naturalistic dashcam images across multiple cities and time periods, showing that our VLM classifications provide strong signal for floods and that our flood detections correlate with known external predictors of flood risk. We then apply BayFlood to practically relevant flood detection tasks using 926,212 images from a single day of flooding in New York City. We show that our flood detections can usefully augment the three aforementioned methods of flood detection and prediction—flood sensors, resident (311) flooding reports, and predicted stormwater accumulation zones. Specifically, BayFlood reveals areas with high predicted flooded fraction that are missed by all of these methods and that are home to 100,150 people; highlights biases in 311 reporting patterns; and suggests locations for new flood sensors, which we have provided to the organization that places the sensors as part of our ongoing conversations. Overall, our research shows how computer vision models, combined with rich dashcam datasets, can usefully complement existing methods to significantly improve urban flooding detection and management. More broadly, we provide a promising general approach for measuring a wide range of phenomena using dense street-level imagery, even when no labels are available.

## Results

### Datasets

Our primary dataset consists of 926,212 dashcam images from a single day of flooding in New York City, with a median of 220 images per Census tract; 94.5% of tracts have at least 50 images. Census tracts are fine-grained geographic areas within the United States with 4000 inhabitants on average; there are 2327 Census tracts in New York City. We conduct our primary analysis at the Census tract level, instead of at the more granular Census block or block group level, to ensure that we have sufficient image coverage for each Census area; 15% of Census block groups and 69% of Census blocks had fewer than 20 images, and 3% of Census block groups and 15% of Census blocks had no images at all.

**Fig. 2:**
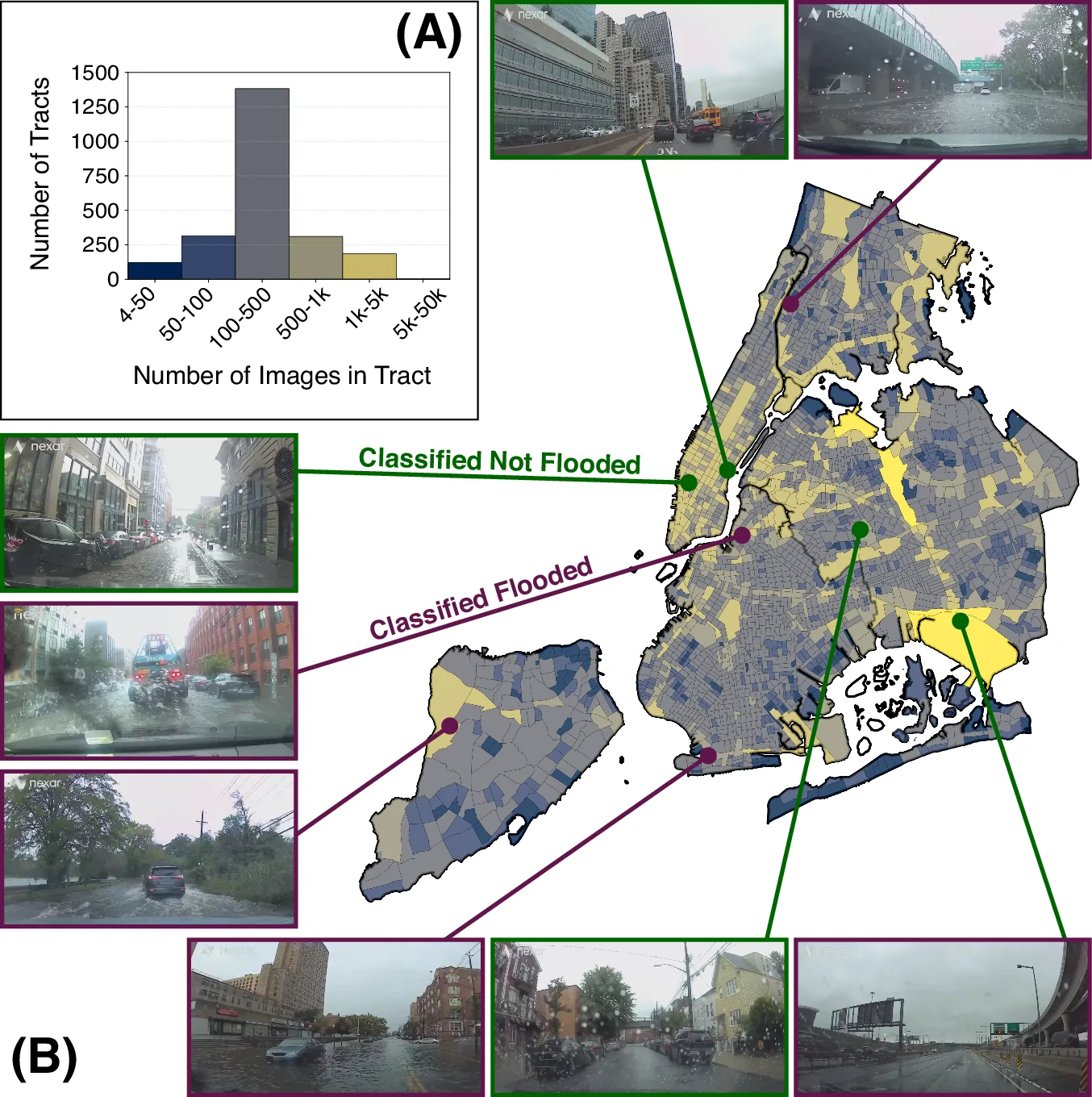
Spatial distribution of images in our primary analysis dataset. **A** Histogram showing the number of dashcam images per Census tract; most Census tracts have from one to five hundred images, and 94.5% of tracts have at least 50 images. **B** Representative images classified as flooded (purple) or non-flooded (green). The color of each Census tract matches the color of a histogram bin in (**A**), and indicates the number of images in that tract.

This dataset was collected during a severe storm in New York City on September 29, 2023 that overwhelmed the city’s drainage and caused widespread flooding and disruption^52–55^ (Fig. 2). The flood triggered a state of emergency in New York City and surrounding areas, severely disrupting transportation (due to subway suspensions, flooded highways, and airport terminal closures), inundating homes and businesses, and requiring dozens of rescues. Occurring just two years after the devastating flooding from Post-Tropical Cyclone Ida, the event disproportionately impacted vulnerable neighborhoods with aging or inadequate drainage, particularly basement apartments housing lower-income residents, and renewed concerns about infrastructure resilience, stormwater management, and protection of at-risk communities.

In preliminary analysis, we observed that it was much harder to reliably detect flooding at night, as the appearance of floodwater is more muted and hard to discern. We therefore do not include nighttime images in our analysis of our primary dataset, removing roughly 13,000 images taken between 7 PM and 6 AM.

We additionally validate our approach using dashcam images from three other days: 158,555 images from New York City on December 17–18, 2023^56^; 331,034 images from New York City on January 9–10, 2024^57^; and 24,383 images from the San Francisco area on February 10, 2024^58^. All dates are chosen because they coincide with storms which caused known flooding events. In total, we validate our flood detection method using 1,440,184 spatiotemporally granular dashcam images collected across multiple dates and cities. This is an important strength of our analysis: to our knowledge, due to the rarity of floods and the difficulty of collecting temporally dense street scene data at the spatial scale of a city, a dataset with these characteristics has not been previously used. Our dashcam images are provided for research purposes by Nexar, a dashcam manufacturer and mobility firm that aggregates image data.

We supplement the aforementioned dashcam datasets and validate our flood detections with additional data sources we fully describe in Supplementary Information Sec. A.2: 311 reports^13^; physical flooding sensors^59^; predictive stormwater accumulation maps^23^; sewer network features like catch basins^60^; digital elevation maps^61^; and American Community Survey (ACS) demographic data^62–66^. We note that some of these data sources provide static measures of flood risk that do not vary across storms—e.g., digital elevation maps and stormwater accumulation maps—but that, in any particular storm, rainfall spatial variability plays a significant role in determining where flooding occurs, motivating the use of real-time data sources like 311 reports and dashcam images.

### Flood detection pipeline

We fully describe our flood detection pipeline in Methods Sec. M.1 and summarize the main steps here. All data sources incorporated in our pipeline are collated in Supplementary Table S1.

Our method for flood detection, BayFlood, has two stages. First, we prompt a vision-language model (VLM), Cambrian-1-13B^48^, to classify whether each dashcam image shows flooding. To classify an image, we provide the model with the image and the following prompt: “Does the street in this image show more than a foot of standing water?” This prompt was chosen because it yielded the best classification performance (see Supplementary Information Sec. B.4) and aligned with the New York City Department of Environmental Protection’s definition of deep flooding^23^. We classify all 926,212 images in our primary dataset; the model classifies 0.2% of images as flooded. Below, we refer to images the model classifies as flooded as “classified positive” and the remaining images as “classified negative.” After classifying all images with the model, we collect ground-truth human annotations for 500 classified positive images, and 500 classified negative images, by manually inspecting the images. This allows us to estimate how often the model is correct, without requiring annotations for the full dataset. We confirm in Methods Sec. M.2 that we achieve similar results with much smaller numbers of ground-truth annotations, indicating that our approach can be used even with very few human labels. Below, we use *y* to denote the manual ground-truth annotation for each image (i.e., whether it truly shows flooding) and $\hat{y}$ as the label from the VLM classifier. Methods Sec. M.1 provides additional details on the VLM classifier.

The second stage of our method applies a Bayesian model to combine the VLM classifications with several additional data sources. Specifically, we use the Bayesian model to estimate the predicted flooded fraction—i.e., the fraction of images in each Census tract *c* that show flooding—by combining several sources of data: the VLM classifications $\hat{y}$; the limited ground-truth annotations *y*; and external data relevant to flood risk (e.g., the number of 311 flood reports in the tract, and the mean elevation in the tract, which can influence flood risk^67^). The Bayesian model provides three benefits. First, it smooths across nearby areas to account for the spatially correlated nature of flooding. Second, it allows us to incorporate external sources of data relevant to flood risk. Finally, it provides principled measures of uncertainty due to both classifier errors and the limited number of labeled images. Overall, the purpose of the Bayesian model is to estimate the proportion of images in each Census area *c* which are truly flooded, *p*(*y* = 1∣*C* = *c*), which we refer to as “predicted flooded fraction” and denote by *r*_*c*_; we use *r*_*c*_ as our primary measure of flood exposure in our analysis. We provide the full mathematical specification of the Bayesian model in Methods Sec. M.1.

### Validation of pipeline performance

We now describe our validation of BayFlood’s performance across multiple cities and timepoints.

First, we show that the VLM classifier displays strong signal for differentiating flooded images from non-flooded images. On our primary dataset, the positive predictive value, $p(y=1| \hat{y}=1)$, is 0.658, indicating that of the images the VLM classifies as flooded, 65.8% are truly flooded; the false omission rate, $p(y=1| \hat{y}=0)$, is 0.006, indicating that of the images the VLM classifies as not flooded, only 0.6% are truly flooded. Put another way, if the VLM predicts an image is flooded, it is 110 × more likely to be flooded. These metrics show both that the classifier clearly provides strong signal for flooding, and that it is imperfect, motivating our use of a Bayesian model to estimate its error rate and incorporate ground-truth annotations. We additionally confirm that our chosen VLM, Cambrian-1-13B, achieved superior zero-shot classification performance when compared to other open-source and proprietary VLMs, and also outperformed a supervised learning approach (Methods Sec. M.1). We discuss additional performance metrics, the motivation for our use of positive predictive value and false omission rate as primary performance metrics, and features of misclassified images, in Supplementary Information Sec. B.2. We assess how well the VLM classifier generalizes to other days and cities by measuring its performance during two other floods in New York City and an additional flood in the San Francisco Bay area (Sec. 2.1). Across all three days, the VLM continues to display strong signal for differentiating flooded and non-flooded images (Supplementary Table S2): images which are classified as flooded are at least 351, 406, and 72 times likelier to be flooded than images which are not. See Supplementary Information Section B.2 for additional details and results.

As a second validation, we assess whether BayFlood’s predictions correlate with known markers of flood risk. Specifically, we measure whether our predicted flooded fraction within each Census area correlates with external markers of flood risk: 311 reports of flooding; presence of FloodNet sensors; predicted stormwater accumulation zones; placement,distribution, and clogging rates of catch basins; and tract elevation. For this analysis, we fit our Bayesian model without incorporating any of these external features, so we are assessing the model’s consistency with external flood risk markers it does not have access to. We define a census tract *c* as “high BayFlood risk” if either *c* ∈ *C*_confirmed_, where *C*_confirmed_ is the set of all tracts with a confirmed ground-truth annotated flood image, or if *r*_*c*_ > *t*, where *t* is the 25th percentile of *r*_*c*_ among all tracts in *C*_confirmed_. (Methods Sec. M.4 justifies this threshold; Fig. S3 replicates results using alternate thresholds, showing that conclusions remain similar.)

We find that high BayFlood risk tracts indeed correlate with external markers of flood risk (Supplementary Fig. S4): they are 1.3 × likelier to have a 311 report and 1.9 × likelier to have a FloodNet sensor. They have 1.3 × fewer catch basins per tract area (although they show no statistically significant difference in clogged catch basin report rates or report resolution times). Their minimum elevation is 2.0 × lower and they have 1.3 × more overlap with shallow stormwater accumulation zones, but no statistically significant difference in overlap with deep stormwater accumulation zones. (All features are statistically significant at *α* = 0.05, *t*-test, except where noted.)

In Methods Sec. M.2 we provide additional validations showing that (1) the Bayesian modeling approach improves predictions; (2) our predictions remain robust even with very few ground-truth annotations; (3) our predictions remain stable even when dashcam data is significantly sparser; and (4) our dataset contains sufficient images to capture spatial variability in flooding within the tract. Collectively, these validations show that our VLM classifications provide strong flooding-relevant signal across multiple cities and timepoints; that our predictions correlate with external flood risk markers; that the Bayesian model further improves predictions; that our approach can be applied even when images or ground-truth labels are more sparse; and that our data can capture within-tract spatial variability in flooding.

### Improving flood detection in New York City

**Fig. 3:**
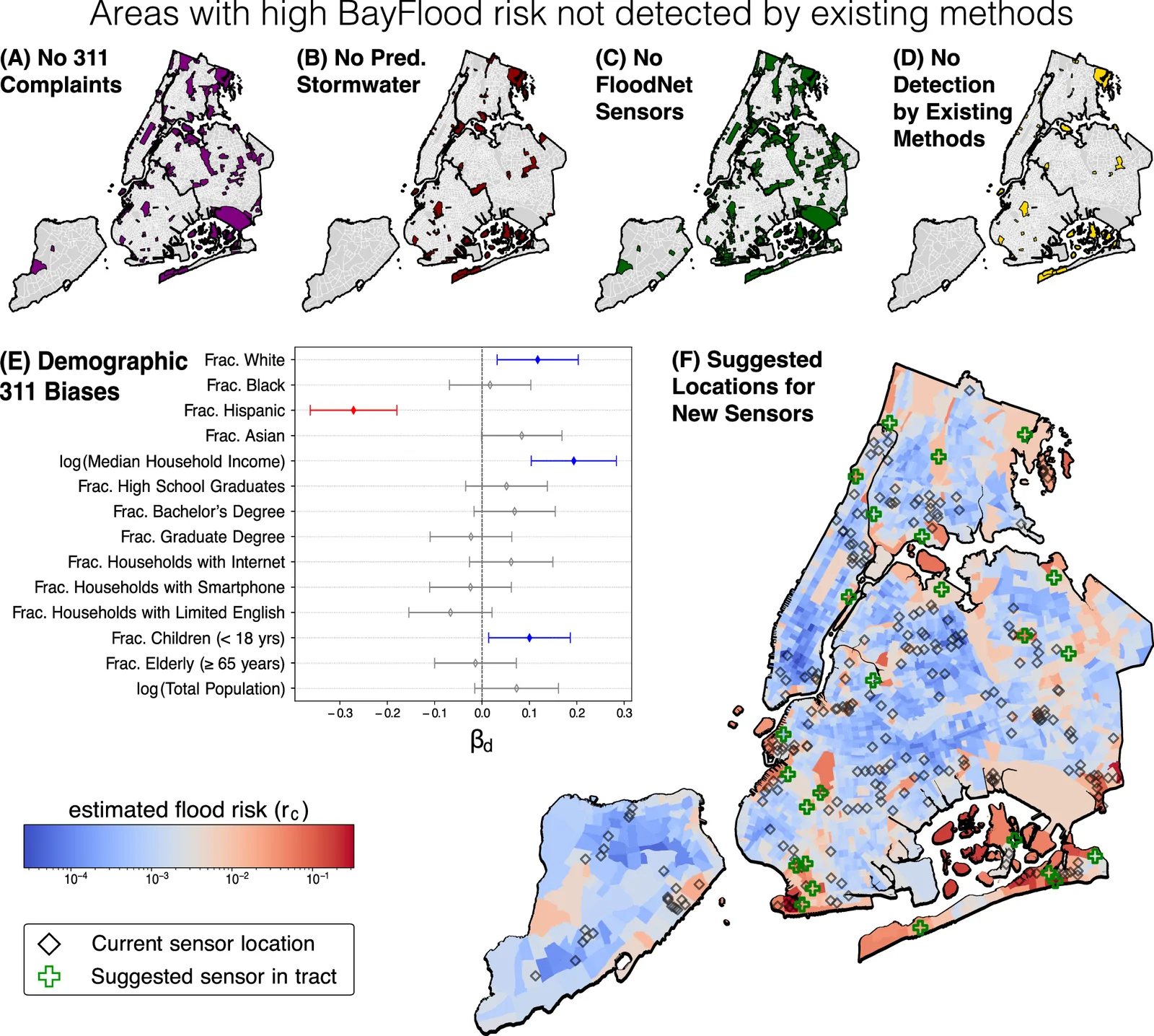
Improving flood detection and response in New York City. Top row (**A–D**): BayFlood detects flooded areas missed by existing methods. From left to right, subplots show Census tracts that are high BayFlood risk, but have no 311 complaints (**A**); no predicted stormwater accumulation (**B**); no FloodNet sensors (**C**); and no coverage from any of these three existing methods (**D**). **E** Demographic biases in 311 reporting rates when controlling for BayFlood-estimated *r*_*c*_. The x-axis plots the coefficients *β*_*d*_ as estimated from the regression specification described in Methods Sec. M.3, along with 95% confidence intervals. Negative coefficients indicate demographic attributes that correlate with lower 311 reporting rates when controlling for predicted flooded fraction *r*_*c*_; positive coefficients indicate demographic attributes that correlate with higher reporting rates. Statistically significant coefficients (*p* < 0.05) are shown in red (negative) or blue (positive); non-statistically-significant coefficients are shown in gray. **F** suggested locations for new FloodNet sensors. Diamonds plot current sensor locations; crosses plot suggested locations for new sensors; and the color of a Census tract shows its BayFlood-estimated predicted flooded fraction.

Having validated BayFlood, we show it can be applied to three important use cases: detecting flooded areas missed by existing methods (Fig. 3A–D); quantifying biases in 311 reports (Fig. 3E); and suggesting new locations for flood sensors (Fig. 3F). These applications are informed by our conversations with government decision-makers as well as with academic-government partnerships like FloodNet. All these analyses are performed on our primary dataset of 926,212 images.

First, we show that our model can identify Census tracts at risk for flooding that are missed by methods currently used by urban decision-makers (Fig. 3A–D). We quantify the number of Census tracts that are high BayFlood risk but do not have a flood-related 311 report, a FloodNet sensor, or predicted stormwater accumulation. (For this analysis, we define tracts with high BayFlood risk as specified above in Sec. 2.3.) We find that 1,046,493 people live in the Census tracts with high BayFlood risk, comprising 12% of New York City’s population. Of these, 832,929 people live in Census tracts with no FloodNet sensors; 254,159 people live in Census tracts with no predicted stormwater accumulation; 390,121 people live in Census tracts with no flooding-related 311 reports; and 100,150 people live in Census tracts with no indicator of flood risk from any of these methods. As an additional robustness check, Supplementary Fig. S2 reports a version of this analysis redefining “high BayFlood risk” tracts as those with at least one ground-truth confirmed flooded image (*y* = 1); this similarly identifies many tracts that are missed by current flood detection methods. Collectively, these results indicate that our model can identify large populations of people who face flooding currently overlooked by some or all of the existing flood detection methods.

As a second use case, we apply our model to quantify potential biases in 311 reporting patterns. Previous work has raised concerns that 311 report distributions may display demographic biases, with some neighborhoods less likely to report incidents when they occur^16,68–70^. We investigate whether BayFlood can quantify these biases. Specifically, for each demographic variable, we conduct a risk-adjusted regression^71^, which measures whether there are demographic disparities in 311 reporting patterns across Census tracts that cannot be explained by disparities in *r*_*c*_. Intuitively, this assesses whether there is inequality in 311 reporting that cannot be explained by inequality in estimated flood exposure. (Methods Sec. M.3 provides a formal mathematical specification of the regression.) Controlling for *r*_*c*_, we find that Census tracts with higher fractions of White residents (*p* = 0.007), lower fractions of Hispanic residents (*p* < 0.001), higher average household incomes (*p* < 0.001), and higher fractions of children (*p* = 0.02) are statistically significantly likelier to have a 311 report (Fig. 3E). These findings accord with past work providing evidence of biases in 311 reporting patterns^16,68–70^, and show that our model can be usefully applied to audit existing methods of flood detection. As an additional robustness check, Supplementary Fig. S5 repeats this analysis controlling for an alternate measure of flood exposure: whether a tract has at least one ground-truth confirmed flooded image (*y* = 1); results are similar.

Our third use case is suggesting new locations for flood sensors. Specifically, based on our finding that many Census tracts have no flood sensors, but high predicted flooded fraction, we provide a proof-of-concept illustration that our model can be applied to identify tracts, which might benefit from the placement of a new sensor.

We assume that if a sensor is placed in a given tract, it can detect a flood in all tracts within a *k*-hop neighborhood, because floods are spatially correlated. We set *k* = 1 in our experiments, but our framework can easily be applied to other *k* (as well as to incorporate additional considerations like the population of a tract, equity in sensor placement, etc). Given the current locations of *T* sensors in a set of Census tracts ${{\mathcal{S}}}^{t}\triangleq \{{c}_{1},{c}_{2},\ldots,{c}_{T}\}$, our task is to place an additional *U* sensors in a set of Census tracts ${{\mathcal{S}}}^{u}\triangleq \{{c}_{T+1},{c}_{T+2},\ldots,{c}_{T+U}\}$ to maximize the sum of *r*_*c*_ in covered areas:

1 $$\sum _{c\in A({{\mathcal{S}}}^{t}\cup {{\mathcal{S}}}^{u})}{r}_{c}$$

where $A({\mathcal{S}})$ denotes all Census tracts in the neighborhood of set ${\mathcal{S}}$. This is a weighted maximum coverageproblem^72^ in which we are given a collection of sets, and our goal is to choose *U* sets such that the weighted sum of elements is maximized. Here, each set is the tracts covered by a sensor in a given location, and the weight for each tract is *r*_*c*_. This problem is NP-hard, but because the optimization objective is submodular, the greedy solution achieves an approximation ratio of $1-\frac{1}{e}$, and is often used. At each iteration, we greedily choose the Census tract that maximizes the sum of *r*_*c*_ in newly covered tracts. We plot the locations chosen by this procedure in Fig. 3F, setting *U* = 25. We have submitted our suggested locations to the FloodNet collaboration^59^ as part of our ongoing conversations with them regarding sensor placements. They expressed interest in our data and methodology as one valuable source of signal to supplement their ongoing placement methodology, which is largely driven by community engagement, stakeholder needs, and equity considerations^73^.

## Discussion

We developed a flood detection approach that applies computer vision to widely available dashcam imagery to identify street flooding, highlighting previously undetected floods and biases in existing urban flood detection methods. Our approach harnesses the ubiquity of dashcam-generated dense street imagery to enhance flooding resilience, providing a cost-effective means to improve detection of flooded areas without the need for extensive manual labeling or sensor deployment. By identifying overlooked flooded neighborhoods, quantifying biases in existing detection methods, and suggesting strategic locations for new flood sensors, our work has the potential to provide urban planners, policymakers, and residents themselves with useful insights into flood management and preparedness, complementing existing detection methods and supporting more targeted interventions and better response planning. Our data sharing with the FloodNet collaboration, which deploys flood sensors across New York City, exemplifies this promise.

Despite its potential, our approach has several important limitations. First, our method is less effective in areas without dense dashcam imagery (e.g., in rural areas, Census tracts are much larger, and fewer images per tract may be available). However, given the proliferation of dashcam use in many cities globally^74^, the scope for application remains substantial. Second, within a city, spatial coverage provided by dashcam images is not uniform, potentially creating biases toward areas with higher vehicular traffic, or with residents who are more likely to utilize ridesharing services^34^. This has the potential to create demographic biases. To investigate this, in Fig. S6 we plot the correlations between Census tract demographic variables, and dashcam image density in the Census tract; while we find some statistically significant correlations (e.g., greater image density in higher-income tracts), all correlations are weak (*R*^2^ < 0.11) suggesting that demographic biases do not strongly skew image coverage. Further, the sheer volume of our dataset—with 94.5% of Census tracts having at least 50 images, and 91.8% of Census tracts having at least one image in every Census block group within the tract—helps ensure that even more sparsely covered neighborhoods have substantial data coverage. Our use of spatial smoothing also mitigates this concern, by enabling us to share information between neighborhoods with little data and spatially adjacent neighborhoods with more data. Our empirical findings also demonstrate that, even with spatially uneven image distributions, our method yields significant improvements in flood detection relative to existing methods.

Third, while we verify that the precision of our approach is high, practitioners should not assume that our approach achieves perfect recall—i.e., that it can identify all flooded areas. This is true for several reasons. First, dashcams cannot capture inundation occurring far from the roadway (e.g., in parks, private properties, or beneath structures, such as bridges that are not visible from the street). They are also less likely to capture roadways that are closed or inaccessible due to deep flooding (though our dataset does contain images from cars stuck in deep floodwater due to flash flooding, and dashcams within parked vehicles can also provide useful images). Second, due to the highly imbalanced nature of the dataset, the recall of our classifier cannot be precisely estimated (Supplementary Information B.2). While this is an important point, many of the applications we highlight do not depend on perfect recall: for example, our method is still able to identify many locations that have flooding that is missed by other methods. Overall, our approach should be viewed as a useful complement to existing methods for flood detection, but one with its own limitations.

A final limitation is that our approach relies on computer vision classifiers, which may produce classification errors; to address this, we empirically validate that our chosen classifier provides strong signal for differentiating flooded and non-flooded images and also outperforms other classification approaches. As computer vision models continue to improve, we would expect the performance of our approach to improve as well, enabling even more accurate and reliable detection of urban flooding incidents.

Looking ahead, our work presents several promising directions for future research. Expanding our analysis to additional locations could help validate the approach in diverse contexts and provide new insights into local flooding patterns. Another promising direction is developing real-time flood monitoring capabilities to support more timely emergency responses; this would likely require deploying more computationally efficient models or edge computing methods directly onto networked dashcams^75^. Dashcam images could also be potentially leveraged to not only detect floods, but to estimate their depth^50,51^; while we explored this direction, we did not have enough ground-truth data on flood depth to validate it, and so leave it as an important direction for future work.

Beyond flood detection, the underlying framework we propose, which leverages the power of zero-shot classifiers to detect objects and incidents from street scenes without requiring large labeled datasets, could be adapted to identify and combat other urban challenges, such as unauthorized or unsafe construction^76^ and untracked street accessibility features^77^; to detect infrastructure of interest, including trees^28–30^, street signs^31^, curb ramps^32^, and manholes^33^; and to measure broader social and urban phenomena, including inequality in policing and surveillance^34,36,78^, demographic variation^37^, infrastructure safety^39^, navigability^40^, and neighborhood change^42,43^. Future work should investigate the potential of our approach in the many other settings in which street scene datasets have proven useful, including urban studies, computational social science, and public health^79–81^.

## Methods

### Flood detection pipeline

Our method for flood detection, BayFlood, has two stages. First, using a VLM, we perform zero-shot classification of whether dashcam images show flooding, and annotate a small number of classified positives and classified negatives with ground-truth human labels. Second, we use the classifier labels $\hat{y}$ and the ground-truth annotations *y* as inputs to a Bayesian spatial model which smooths across adjacent areas and incorporates features relevant to flood risk. The raw images are not used as inputs to the Bayesian model.

#### Zero-shot vision-language model classification

We perform zero-shot classification of whether each image is flooded using the Cambrian-1 model^48^, an open-source VLM developed in 2024, which achieved state-of-the-art results among open-source models like LLaVA-NeXT^82^, and comparable performance to the best proprietary models, including GPT-4V^83^. We use the 13B-parameter version of the model, and use the prompt “Does the street in this image show more than a foot of standing water?" This prompt was the most performant on the annotated inspection set we describe below, and aligns with the definition of ‘deep’ flooding as defined by the New York City Department of Environmental Protection^23^. We use the 13B-parameter version of Cambrian-1 because it considerably outperforms the smaller 8B-parameter version (Supplementary Table S3) without introducing the significantly higher inference costs of the 34B-parameter version. Performing inference on all 926,212 images in our primary dataset takes approximately 18 h when distributed across 6 Nvidia RTX A6000 GPUs. The model classifies 0.2% of images as flooded, consistent with the imbalanced nature of the dataset.

#### Measuring model performance

We assess Cambrian-1-13B’s performance on our primary dataset by randomly sampling 500 images classified as positive and 500 classified as negative, and manually annotating them. One researcher from the team annotated all images to ensure annotation criteria were consistent. Each image was annotated as positive if it showed definite flooding: namely, the image contained a view of the street in front of the vehicle—i.e., the dashcam itself was positioned at an angle suitable for capturing the street—and the street showed significant flooding. Ambiguous images, and ponding and other small puddles, were marked as negative.

We quantify model performance by reporting the positive predictive value $p(y=1| \hat{y}=1)$ (i.e., the proportion of classified positives which are truly positive) and the false omission rate $p(y=1| \hat{y}=0)$ (i.e., the proportion of classified negatives which are truly positive). We also validate model performance on three additional dashcam image datasets from other dates and cities (Sec. 2.1).

#### Assessment of annotator reliability

We evaluate the reliability of our ground-truth human annotations by having two additional annotators separately re-annotate the same images (after discussing annotation criteria with both annotators) and evaluating collective agreement. The two additional annotators annotate a random subset of 400 images from the original annotation set of 1000 images. Agreement between all three annotators is very good: Cohen’s *κ* between the first and second annotator is 0.84; Cohen’s *κ* between the first and third annotator is 0.96; and Fleiss’s *κ* between all three annotators is 0.88. All of these values indicate “strong” or “almost perfect” agreement between annotators, indicating the annotations are reliable enough for use^84,85^.

#### Comparison to classification baselines

We compare the zero-shot classification performance of Cambrian-1-13B to that of several other open-source VLMs—Cambrian-1-8B, CLIP, and DeepSeek Janus-Pro-7B. We also compare to a supervised learning baseline (a ResNet fine-tuned on a subset of the dataset with flooding labels). We fully describe these baselines in Supplementary Information Sec. B.3. For all models, we compare performance using the same metrics discussed above—namely, for each model, we estimate $p(y=1| \hat{y}=1)$ and $p(y=1| \hat{y}=0)$ by taking a random sample of its positive classifications, and a random sample of its negative classifications, and annotating with ground-truth labels. Supplementary Table S3 shows that our chosen model (Cambrian-1-13B) outperforms all classification baselines, achieving higher $p(y=1| \hat{y}=1)$ (*p* < 0.001, t-test), and lower but comparable $p(y=1| \hat{y}=0)$ (differences not statistically significant, *t*-test). In Supplementary Information Sec. B.3 we describe additional comparisons to a proprietary closed-source model (GPT-4o-mini), which was much more expensive to run and produced inferior results. Collectively, these results show that our preferred model, Cambrian-1-13B, achieves superior performance to other image classification methods.

#### Comparison to alternate prompts

We compare our preferred prompt to four prompts used in prior work^49–51^. We find that our preferred prompt both yields better classification performance and faster inference; see Supplementary Information Sec. B.4 for additional details.

##### Bayesian modeling of vision-language model classifications

After classifying all images in our primary dataset using the VLM and manually annotating a small subset of the classified images, we then fit a Bayesian model on the 926,212 model classifications (positive or negative), manual ground-truth annotations (positive, negative, or unknown), and additional flood-relevant features. Because we only annotate 1000 images, the vast majority of annotations are unknown. The raw images are not used as inputs to the Bayesian model.

The purpose of the Bayesian model is to estimate the proportion of images in each Census area *c* which are truly flooded, *p*(*y* = 1∣*C* = *c*), while accounting for uncertainty due to classifier error and finite samples of images; smoothing across adjacent areas; and incorporating external data relevant to flood risk.

We now describe the observed data for the Bayesian model. Let *y* denote the manual ground-truth annotation for each image (i.e., whether it is truly flooded) and $\hat{y}$ its label from the VLM classifier. In each Census area, we have images of six types, depending on (a) whether the image’s classifier label is positive or negative and (b) the ground-truth annotation label is positive, negative, or unknown (2 possibilities × 3 possibilities = 6 image types). Thus, our observed data for each Census area *c* consists of a set of six numbers: the counts of images in the Census area ${n}_{\hat{y={\ell }_{\hat{y}}},y={\ell }_{y}}^{(c)}$ where the classifier label is ${\ell }_{\hat{y}}\in \{0,1\}$ and the ground-truth label is *ℓ*_*y*_ ∈ {0, 1, ?}. For example, the observed data for one Census area with 100 images might be “90 images were classified negative, and have unknown ground-truth label; nine were classified positive, and have unknown ground-truth label; and one was classified positive, and has a positive ground-truth label.” For each Census area *c* we additionally observe a vector *X*_*c*_ of flood-relevant features—for example, whether the area is a flood risk zone or has any resident complaints of flooding (see Supplementary Information Sec. A.2 and Sec. C.2 for full details).

We now summarize the mathematical details of the Bayesian model (providing additional details in Supplementary Information Sec. C). Our main quantity of interest is the probability that an image in a Census area *c* shows flooding, *p*(*y* = 1∣*C* = *c*). We model this as follows:

2 $$p(y=1| C=c)={\mathrm{logit}}^{-1}(\alpha+{X}_{c}\beta+{\phi }_{c}\cdot {\sigma }_{\phi })$$

where *α* is an intercept term, *β* is the feature coefficients, and *ϕ*_*c*_ is an Intrinsic Conditional Auto-Regressive (ICAR) spatial component which varies by Census area, a standard technique to capture spatially correlated phenomena like flooding^86,87^ by smoothing across adjacent areas.

We model VLM classifier errors by introducing parameters to capture the classifier’s true positive rate ${\theta }_{\hat{y=1| y=1}}\triangleq p(\hat{y}=1| y=1)$ and false positive rate ${\theta }_{\hat{y=1| y=0}}\triangleq p(\hat{y}=1| y=0)$. We assume these error rates remain constant across Census areas.

The log likelihood (LL) of the observed data in Census area *c* is:

3 $$\underbrace{\sum _{{\ell }_{\hat{y}}=0,1}\sum _{{\ell }_{y}=0,1}{n}_{\hat{y={\ell }_{\hat{y}}},y={\ell }_{y}}^{(c)}\,\mathrm{log}\,p(\hat{y}={\ell }_{\hat{y}},y={\ell }_{y}| C=c)}_{\,\mathrm{LL}\,\,\,\mathrm{of}\,\,\,\mathrm{images}\,\,\,\mathrm{with}\,\,\,\mathrm{ground}-\mathrm{truth}\,\,\,\mathrm{labels}\,}+$$

4 $$\underbrace{\sum _{{\ell }_{\hat{y}}=0,1}{n}_{\hat{y={\ell }_{\hat{y}}},y=?}^{(c)}\,\mathrm{log}\,p(\hat{y}={\ell }_{\hat{y}}| C=c)}_{\,\mathrm{LL}\,\,\,\mathrm{of}\,\,\,\mathrm{images}\,\,\,\mathrm{without}\,\,\,\mathrm{ground}-\mathrm{truth}\,\,\,\mathrm{labels}\,}$$

We can write $p(\hat{y}={\ell }_{\hat{y}},y={\ell }_{y}| C=c)$ and $p(\hat{y}={\ell }_{\hat{y}}| C=c)$ in terms of *p*(*y*∣*C* = *c*) and the error rates *θ*, allowing us to express the LL of the observed data as follows:

5 $$p(\hat{y}=1,y={\ell }_{y}| C=c)=p(y={\ell }_{y}| C=c)\cdot {\theta }_{\hat{y=1| y={\ell }_{y}}}$$

6 $$p(\hat{y}=0,y={\ell }_{y}| C=c)=p(y={\ell }_{y}| C=c)\cdot \left(1-{\theta }_{\hat{y=1| y={\ell }_{y}}}\right)$$

7 $$p(\hat{y}={\ell }_{\hat{y}}| C=c)=p(\hat{y}={\ell }_{\hat{y}},y=1| C=c)+p(\hat{y}={\ell }_{\hat{y}},y=0| C=c)$$

To complete the Bayesian model specification, we place weakly informative priors over all model parameters. We fit the model using Hamiltonian Monte Carlo (HMC)^88,89^ as implemented in the probabilistic programming language Stan^90^. Below, we will use *r*_*c*_ ≜ *p*(*y* = 1∣*C* = *c*) as shorthand to refer to the model’s predicted flooded fraction in a given Census tract.

### Additional validations of flood detection pipeline

In the main text, we describe the two primary validations performed on our model (Sec. 2.3), showing that the VLM classifier provides a strong signal for detecting flooded images, and the predicted flooded fraction *r*_*c*_ correlates with known markers of flood risk. Here we describe four additional validations, showing that (1) the Bayesian model improves out-of-sample prediction; (2) model predictions remain stable even with very few ground-truth annotations; (3) model predictions remain stable even with sparser image data; and (4) we have good ability to capture spatial variability at the Census tract level.

#### Bayesian modeling improves out-of-sample prediction

We show that our Bayesian approach improves predictions of where flooded images will occur on a held-out test set, relative to both simple heuristics (e.g., the fraction of images which are classified as positive by the VLM) and machine learning baselines.

We perform this validation as follows. After classifying the 926,212 images in our primary dataset with the VLM, we partition them into a train set (which we use to fit the Bayesian model and the baselines on the classifications) and a test set (which we use to assess out-of-sample performance). We use three metrics to assess predictive performance at the Census tract level: (1) Pearson correlation with fraction of images in the tract which are classified flooded; (2) AUC for predicting whether the Census tract will have any classified flooded images; (3) AUC for predicting whether the Census tract will have any ground-truth annotated flooded images. To minimize the noisiness of these metrics on the test set, we reserve 70% of the dataset for the test set. Thus, the train set for this validation consists of the VLM classifications $\hat{y}$, and ground-truth annotations *y*, for 30% of the images; the test set consists of the VLM classifications and ground-truth annotations for the remaining 70%.

We compare to three sets of baselines. First, we compare to several heuristic baselines (i.e., simple functions of the VLM classifications or ground-truth annotations which do not require machine learning): (1) the fraction of train set images in a Census tract which are classified positive by the VLM; (2) the number of train set images which are classified positive; (3) whether any train set images are classified positive; (4) whether any train set images are ground-truth annotated positive; and (5) the number of train set images which are ground-truth annotated positive. Second, we compare to supervised learningbaselines which are trained on the train set to predict the fraction of images which are classified as flooded, and the number of ground-truth annotated flooded images, from the same set of flood-relevant features our Bayesian model uses (Supplementary Information Sec. C.2). We fit both linear regression and random forest models. Finally, we compare a graph smoothing baseline, which applies Laplacian smoothing using the Census tract adjacency matrix. We fully describe all baselines in Supplementary Information Sec. C.3.

Our Bayesian model outperforms all baselines on all considered metrics (Supplementary Table S5), demonstrating it provides benefit over alternative ways of aggregating the VLM annotations.

#### Model predictions remain stable even with very few ground-truth annotations

Because ground-truth human annotations can be expensive to produce in some settings, we investigate whether the predicted flooded fractions of our Bayesian model, *r*_*c*_, remain stable even with very few ground-truth annotations, showing that the model can be reliably applied even when annotations are sparse. Specifically, we refit the Bayesian model on datasets where the number of ground-truth annotations have been downsampled by a factor of 2 × to 10 × ; 10 × downsampling corresponds to only 50 annotated positives and 50 annotated negatives. We find that the model’s predictions on these downsampled datasets remain highly correlated with predictions on the full dataset (between 0.97 and 0.99 across all downsampling ratios). This suggests that our Bayesian approach can be applied even in settings where very few ground-truth annotations can be collected. Further, because the Bayesian model yields measures of uncertainty on all estimates, it naturally provides principled estimates of the stability of model predictions, guiding the collection of additional annotations if needed.

#### Model predictions remain stable even with sparser image data

In some empirical settings, fewer images may be available (if, for example, there are fewer drivers with dashcams). We thus investigate whether the predictions of our Bayesian model, *r*_*c*_, remain stable under random downsampling of the entire flooding dataset of 926,212 images. Specifically, similar to the validation above, we refit the Bayesian model on datasets where the number of images has been downsampled by a factor of 2 × to 5 × . Because we apply this downsampling to all the images, not just to the annotated ones, we would expect this to have a more significant effect on model performance. We find that predictions remain strongly correlated with the original predictions even under substantial downsampling (correlations of 0.95, 0.83, and 0.84 for 2 × , 4 × , and 5 × downsampling, respectively). At higher downsampling ratios the sampler no longer reliably converges (some parameters exceed an $\hat{R}$ threshold of 1.1), so we restrict this analysis to the ratios at which all parameters converge. Collectively, these results demonstrate that our model predictions remain stable even when image data is significantly sparser.

#### Capturing spatial variability within census tracts

A potential concern is that if a tract contains too few images, or if images are concentrated in a small area within the tract, then localized flooding might go undetected. To investigate this, we examine whether the number of images per tract in the Nexar dataset is sufficient to capture spatial variability in flooding within those tracts.

Fortunately for this purpose, the large majority of tracts contain substantial numbers of images, in particular, 99.4% of tracts have at least ten images, 98.7% have at least 20, 94.5% have at least 50, and 81.0% have at least 100. To assess whether this is sufficient to capture spatial variability in flooding, we conducted a power analysis as follows: we simulated a world in which each image in the tract had a probability *p* of being truly flooded, and computed the probability that our data would contain at least one flooded image for the tract. In other words, we assess whether we have enough images in most tracts that we have a good chance of having at least one image that shows flooding if it is in fact occurring. (Mathematically, we computed the probability of detecting at least one flooded image as 1 − (1−*p*)^*N*^, where *N* is the number of images in the tract and *p* is the proportion of images that are flooded). This analysis showed that we have a 90% chance of having a flooded image in 99.7% of Census tracts that are 50% flooded; in 99.5% of tracts that are 25% flooded; and in 98.5% of tracts that are 10% flooded. This demonstrates that we are quite likely to be able to identify flooding in the vast majority of tracts that are truly significantly flooded.

As an additional corroborating analysis, we confirmed that within Census tracts, the images we have are not all confined to the same area (which would reduce our ability to detect flooding across the entire tract). 91.8% of Census tracts had at least one image in every nested Census block group the tract contained, demonstrating that in the vast majority of tracts, the images provide good spatial coverage of regions throughout the tract. Cumulatively, these analyses demonstrate that we have good ability to capture spatial variability at the Census tract level.

### Risk-adjusted regression for exploring 311 report rate biases

We use risk-adjusted regression^71^ to quantify biases in 311 reports. For each demographic variable, we perform a separate univariate logistic regression to assess whether there are demographic disparities in 311 reporting patterns across Census tracts which cannot be explained by our model’s predicted flooded fraction in a tract:

8 $$p(\,\mathrm{Tract}\,\,c\,\,\mathrm{has}\,\,311\,\,\mathrm{report})={\mathrm{logit}}^{-1}(\gamma+{\beta }_{r}{r}_{c}+{\beta }_{d}{d}_{c})$$

where *γ* is an intercept term; *r*_*c*_ is our model’s predicted flooded fraction in tract *c*; *d*_*c*_ is a demographic feature (e.g., the fraction of the tract which is white); and the *β*s are the regression coefficients. Fig. 3E plots the estimated demographic coefficients *β*_*d*_ from the univariate regressions. (All demographic data comes from the American Community Survey 2023 5-Year Estimates.).

### Thresholding high BayFlood risk threshold, *r*_*c*_

We chose to define “high BAYFLOOD risk” by setting the threshold for *r*_*c*_ using the 25th percentile of tracts with confirmed flooding for two reasons. First, this threshold captures most of the tracts that likely truly had flooded images (because 75% of tracts with known flooded images were above this threshold). Second, setting an unduly low threshold would increase vulnerability to outliers (as would be a risk if, e.g., we used the 1st percentile of tracts with confirmed flooded images).

## Supplementary information


Supplementary Information
Transparent Peer Review file


## Data Availability

United States Census and American Community Survey datasets used in our analysis are available online^62–66^. The New York City Department of Environmental Protection’s stormwater flooding maps and the digital elevation map are available online^61,91^. FloodNet sensor locations may be requested via the FloodNet data access request form^92^. The raw Nexar dashcam images cannot be made publicly available because they are proprietary; data access may be requested by contacting Nexar, Inc. However, we provide aggregated data for replicating our analyses, including the VLM classifications of the images, at this GitHub repository.

## Code availability

Our Bayesian model and additional code to reproduce our results is available at this GitHub repository.
