# Supplementary material for: Improving flood detection with large-scale dashboard camera data
Source: Nat Commun. 2026 Jul 29;17:9208. doi: 10.1038/s41467-026-75938-1 (PMC13522533; doi:10.1038/s41467-026-75938-1)
Supplement: Supplementary file 1 [file 41467_2026_75938_MOESM1_ESM.pdf]

Supplementary Information

A Details of empirical setting

A.1 Privacy protections for dashcam dataset

Our use of this dataset has been previously deemed not human subjects research by our institution's IRB, as our data depicts public street scenes and we do not analyze pedestrians. We have also examined the privacy implications of dense street imagery through the lens of contextual integrity [78], finding that pedestrian and group-related analysis tasks can carry privacy harms; this project avoids harm by focusing solely on the physical environment. We are committed to ethical use of our data, and our data provider maintains a high data anonymization standard of blurring pedestrians, license plates, and dashboards prior to us having any access. The data provider additionally blacks out the top and bottom of each image to remove any personally-identifiable information from the driver that may appear on the vehicle dashboard.

A.2 Additional dataset information

Nexar dashcam data

The primary input to our method is dashcam images of public street scenes [35]. Consumer, vehicle-mounted dashcams, known for their utility in safety and liability protection, provide spatially and temporally dense image data, capturing the urban *streetscape*. Relative to prior datasets, like Google Street View, dashcam datasets offer much higher temporal density, rendering them superior for analyzing short-horizon events like flooding; in contrast, the gap in time between consecutive images in Google Street View can be as large as 7 years [93]. Our dashcam dataset is provided by Nexar, whose data has been widely used in prior work [34, 35, 76, 94–96]. Nexar images are 1280 × 720 pixels and are captured from cameras affixed to the windshield of actively-driving vehicles, mostly those of ridesharing[1] drivers.

A.2.1 External covariate datasets

We make use of the following external datasets relevant to flood risk to contextualize and validate our flood detections.

311 reports

Crowdsourced resident reporting systems like NYC311 have emerged as important indicators of infrastructural problems, including street flooding. In a typical 311 system, residents have the ability to submit reports of non-emergency problems (via app, internet, or phone) which are then routed to the appropriate city agency for remediation [12]. During the September 29, 2023 storm in New York City, for example, there were 2,171 calls made to 311 pertaining to flooding-related issues (see Supplementary Information §A.3 for the list of issues we define as flooding-related). While 311 provides valuable information on potential floods, and is thus a useful external validation of our model's flood detections, it is also known to contain biases due to disparities in how likely neighborhoods are to report problems [16, 69, 70, 97–99]. We show our approach can be applied to audit these biases.

Physical flooding sensors

Physical flooding sensors are an important current source of flooding signal for cities [100]. We rely on data from FloodNet [59], a New York City government-academic partnership to develop low-cost, easy-to-assemble flooding sensors and install them throughout high-risk areas. At the time of the flood on which we conduct our primary analysis (September 29, 2023) there were 67 active FloodNet sensors; as of December 12, 2024 (our last data update), there are 253 unique FloodNet sensors placed at some point in time. We use the locations of these 253 sensors as an indicator of flood risk. Placement decisions are informed by a detailed, community-informed process [101].

[1] Ridesharing refers to services that offer on-demand passenger pickup and dropoff at a chosen destination; companies that offer ridesharing include Uber and Lyft.

Stormwater accumulation maps

Stormwater accumulation maps have been developed as a useful tool for governments and citizens to facilitate flood readiness [102–104]. We note that, in contrast to our flood detection method, these maps are not designed to detect flooding during specific events; rather, they are static maps designed to predict stormwater accumulation under a given amount of rainfall and sea level rise.

We use Stormwater Flooding Maps from the New York City Department of Environmental Protection (NYC DEP) [105], which use simulations that incorporate drainage system data and flow capacity measurements to provide estimates of (1) shallow flooding (more than 4 inches, less than 1 foot) and (2) deep and contiguous flooding (greater than 1 foot). The maps we use were developed under the assumption of a fully functioning drainage system and with catch basins free of debris [23, 106]. NYC DEP has produced maps of four weather scenarios: (1) a limited stormwater flooding event (peak ≈ 1.77 inches rainfall/hour) without sea level rise, (2) a moderate stormwater flooding event (≈ 2.13 inches/hour) without sea level rise, (3) a moderate stormwater flooding event (≈ 2.13 inches/hour) with 2050 sea level rise, and (4) an extreme stormwater flooding event (≈ 3.66 inches/hour) with 2080s sea level rise. In our analysis, we use scenario (2), a moderate stormwater flooding event (≈ 2.13 inches/hour) without sea level rise, because the September 29 storm we analyze was a 100-year storm where about 2 inches of rain fell in one hour in Central Park [107].

Comparing flooding zones (polygons) to flooding in dashcam images (represented as points), introduces the methodological challenge of comparing areal data to point observations. We address this as follows. In our analysis in Figure 3B, we assess how many tracts are high BayFlood risk (a metric computed at the tract level) but have no intersection with a predicted stormwater zone. In our analysis in §2.3, we compute the fraction of the tract which overlaps with a shallow stormwater accumulation zone and the fraction of the tract which overlaps with a deep stormwater accumulation zone, and use those as covariates.

Catch basins

Catch basins are a key part of a high-performing sewage network; they prevent flooding by directing stormwater away from streets and properties. Catch basins must be cleaned regularly, as clogging can severely exacerbate street flooding. We use data on both the physical distribution of catch basins in the city [60] and *311 reports* of clogged catch basins [13] to create three covariates at the Census tract level: (1) catch basin density; (2) historical presence of any clogged catch basin complaint (from June 1 2023 to September 29 2023); and (3) average clogged catch basin report resolution time during the historical period.

Digital elevation maps (DEM)

We utilize New York City's Digital Elevation Map [61] to compute basic elevation metrics for each Census tract in the city, including minimum elevation, mean elevation, and maximum elevation. The DEM was generated with one-foot granularity using LiDAR data, and is meant to ground elevation in feet above sea level, with all built surface features removed.

American Community Survey (ACS) demographic data.

We use the US Census' American Community Survey to investigate our model's population coverage and investigate biases in 311 data. Following similar work [16, 69, 70], we select datasets on total population, reported race and age [62], household income [65], educational attainment [63], access to technology [66], and language spoken at home [64].

A.3 Additional data processing details

Census tract adjacency matrix

Our Bayesian model makes use of a Census tract adjacency matrix for spatial smoothing. We describe our approach in assessing adjacent Census tracts in New York City, as the city's topography and dense development have produced intricate Census geographies. We use the water-clipped version of the 2020 NYC Census tracts, provided by the NYC Department of City Planning [108]. Using tracts with water areas included creates inaccurate adjacency relationships, such as tracts in downtown Manhattan being neighbors to Staten Island. To compute adjacency, we buffer (expand) each tract by 500 feet, and then define the two tracts as adjacent if these expanded boundaries intersect. A buffer threshold of 500 feet is necessary to capture neighboring tracts across the East River. Thus,

our measure of adjacency should be regarded as capturing spatial proximity between two tracts (as opposed to them necessarily sharing a border), consistent with our purpose in using the adjacency matrix for spatial smoothing. Supplementary Figure S1 depicts the resulting adjacency matrix.

311 reports

We classify the following complaint types as flooding-related for the purposes of our analysis: sewer backup, street flooding, catch basin clogged/flooded, manhole overflow, and highway flooding. We include reports from the entire day of September 29, 2023.

Elevation data

We downsample the 1-foot Digital Elevation Model of NYC by a factor of 10 because it accelerates performance without significantly compromising accuracy at the granularity of a Census tract.

B Additional VLM details

B.1 Cambrian-1

We employ Cambrian-1-13B [48] as our primary vision-language model for flood detection from dashcam imagery. Cambrian-1 prioritizes accuracy rather than inference throughput; this aligns with our use case, where we retroactively analyze dashcam imagery offline, and thus do not need maximum inference efficiency.

B.2 Measurement of VLM performance

Annotation procedure

For all VLMs, including baseline models, we measure performance on our primary dataset by manually annotating a random subset of classified positives and a random subset of classified negatives. For our preferred VLM, we additionally assess performance on three additional days (as described in §2.1) by manually annotating 250 randomly-sampled classified negatives and 250 randomly-sampled classified positives (or, in cases where there are fewer than 250 classified positives, all classified positives) – see Supplementary Table S2.

We only annotate an image as flooded if it unambiguously shows flooding. In very rare cases, technical artifacts render this ambiguous, including cases where (a) the view of the forthcoming street is obscured by the vehicle dashboard or (b) the dashcam is mispositioned, producing images that do not depict any part of the street; we mark these as negative. We similarly annotate images with visually ambiguous flooding (e.g., ambiguous reflections from sunlight) as negative.

Choice of performance metrics

Our primary metrics for measuring performance are positive predictive value ($p(y = 1|\hat{y} = 1)$) (also known as precision) and false omission rate ($p(y = 1|\hat{y} = 0)$) (equivalently, one minus the negative predictive value). Diverse metrics of classification performance are used in flood detection settings [109–112]; we choose to focus on these metrics for two reasons. First, they are widely used measures of predictive performance, including in flood modeling contexts [110–112]. Second, our setting is highly *class-imbalanced*: very few images truly show flooding, and the vast majority of classifications will be negative. In these settings, precise estimation of many measures of prediction performance is very challenging. However, the metrics we choose can be precisely estimated by a straightforward procedure: choosing a random set of classified positives and annotating them (yielding $p(y = 1|\hat{y} = 1)$) and a random set of classified negatives and annotating them (yielding $p(y = 1|\hat{y} = 0)$).

In contrast, in class-imbalanced settings, precisely estimating other standard metrics of prediction performance, including recall (and thus F1 score, which includes recall as an input, along with related metrics like critical success index) is very difficult. This is because recall — $p(\hat{y} = 1|y = 1)$ — conditions not on $\hat{y}$, which can be easily computed for all images, but on y, which requires expensive manual annotation. In class-imbalanced settings, when $p(y = 1)$ is very small, it is infeasible to annotate enough images to produce a sufficiently large sample of images with $y = 1$ for reliable estimation of recall. An alternative is to estimate recall using Bayes' rule:

$$p(\hat{y}=1|y=1) = \frac{p(y=1|\hat{y}=1)p(\hat{y}=1)}{p(y=1|\hat{y}=0)p(\hat{y}=0)+p(y=1|\hat{y}=1)p(\hat{y}=1)}$$

but this also yields extremely noisy estimates of recall. The reason is that almost all the images in the dataset are classified as negative ($p(\hat{y}=0) \approx 1$ and $p(\hat{y}=1) \approx 0$); thus, even tiny fluctuations in the estimate of the false omission rate, $p(y=1|\hat{y}=0)$, yield very large changes in the denominator, and thus in the estimate of recall. For example, on our primary dataset, even though our estimated false omission rate is tiny (0.006), our 95% confidence interval for our estimate of recall is very wide: 0.07 - 1.00. Similarly, our 95% confidence interval for F1 score is 0.13 - 0.79. Both these intervals are so wide as to be essentially uninformative. Similarly, our estimates of recall vary significantly across different cities, because on our three validation datasets, there are no false negatives in our annotation sample, yielding very high estimates of recall.

In contrast, our preferred metrics are much more precisely estimated: for example, on our primary dataset, our 95% confidence interval for positive predictive value is 0.62 - 0.70, and 95% confidence interval for the false omission rate is 0.00 - 0.01. This motivates our focus on these metrics.

Features of misclassified images

One challenge in the street flooding detection task is that the appearance of standing water varies greatly under different lighting conditions and on different surfaces. In our inspection set of 1000 images from September 29, 2023, false negatives (3 images out of 500 random classified non-flooded images) occur less frequently than false positives (171 images out of 500 random classified flooded images) due to the low overall rate of flooding. We separately inspect both false positives and false negatives to look for shared features that may contribute to misclassification.

Among the 171 false positives, the following features occur repeatedly and may contribute to misclassification: (1) windshields reflecting objects (e.g., a book, bag, or flag) placed atop the vehicle dashboard; (2) bright sunlight reflections on slick pavement; (3) stripes of water droplets across most or all of the windshield; (4) mud on the side of a highway; (5) cobblestone streets or torn-up pavement; (6) high-visibility orange road work fixtures; (7) emergency vehicles from FDNY or NYPD; (8) steam rising from a manhole; and (9) static and other visual artifacts from the networked data transmission process. Among the 3 false negatives, the street scene is cloudy, and the pavement and standing water are dark and blend together. Without careful scrutiny, the street and the standing water appear as one continuous surface.

Generalization across cities and time periods

To assess generalization across cities and time periods, in Table S2 we report classification performance across 3 additional days following flooding (two in New York City and one in San Francisco). Across all three days, the VLM continues to display strong signal for differentiating flooded and non-flooded images: images which are classified as flooded are at least 351, 406, and 72 times likelier to be flooded than images which are not. In our three validation datasets, we do not observe any false negatives among the images classified negative. We thus compute these numbers using an upper bound of one false negative. The lack of false negatives amongst the validation datasets is likely due to the fact that the storms that occurred on those days were less severe, and also occurred the night before the date of image capture, leading to less pervasive flooding, fewer flooded images, and fewer false negatives. The lower rate of positive images also helps explain why the estimated PPV in San Francisco is relatively low — we estimate that the true fraction of positive images that day is only 4×10^{-5}, producing a lower PPV as well. (PPV in San Francisco is also more noisily estimated because the overall dataset is much smaller, potentially leading to imprecise estimates.) Overall, while our classifier continues to strongly differentiate flooded and non-flooded images on our three validation datasets, these nuances highlight the importance and the challenge of assessing generalization across multiple cities and time periods — an important direction for future work.

B.3 VLM baselines

We compare the performance of our preferred VLM (Cambrian-1-13B [48]) to several alternate open-source VLMs with zero-shot prompting: CLIP [46]; DeepSeek's Janus Pro VLM [113]; and Cambrian-1-8B [48]. For all baselines, we experiment with multiple prompts and report the highest-performing configuration for each VLM in Supplementary Table S3. Our preferred model achieves superior performance to the baselines. We estimate performance of each baseline by sampling a

random subset of 250 classified positives, and 250 classified negatives, and obtaining ground-truth manual annotations.

We also compared our zero-shot prompting method to a supervised learning approach using noisy labels. Specifically, we first used CLIP (zero-shot) to identify candidate flooded images; then used GPT-4V (zero-shot) to further filter down the set, producing a set of noisy positives; and then fine-tuned a ResNet to distinguish between the noisy positives and all other images. (We did not use GPT-4V to annotate all images because it would impose a prohibitive cost on a dataset of our size.) We found that this method achieved inferior performance to our preferred approach (Supplementary Table S3) at the cost of considerable additional complexity, and thus did not pursue it further.

Finally, we compared our preferred VLM to a strong closed-source model — OpenAI's GPT-4o-mini — to confirm that our preferred VLM was not producing far inferior performance. Because using GPT-4o-mini to annotate all images would be prohibitively expensive, we instead evaluated its performance on a random sample of 10% of the images in our primary dataset, yielding 92,621 images. We found that GPT-4o-mini did not perform well with either of the prompts that worked well for other models; it classified only 9 of the 92,621 images as flooded, a far lower rate of flooded images than we identified with other models and corroborated with ground-truth annotation. Due to this inferior performance, as well as the prohibitive cost and difficulty of reproducing results with closed-source models, we used open-source VLMs for our primary results.

B.4 Prompt Engineering

For each VLM we test, we assess multiple prompts and report results from the one which yields optimal performance. For the VLM we used for our primary analysis, Cambrian-1-13B, we compared performance of six prompts. First, we measured the performance of two short prompts: (a) *Does the street in this image show more than a foot of standing water?* and (b) *Does this image show a flooded street?* We found that the latter prompt classified many more images as positive, resulting in slightly higher recall but much lower precision, and thus favored the former. Second, we measured the performance of four more complex prompts used in prior work [49–51] (full prompt text provided in Supplementary Information §D). Supplementary Table S4 summarizes the results of this analysis. Only one of the four prompts [49] performed comparably to (but somewhat worse than) our original prompt; the three other prompts, which suggested the usage of contextual environmental cues and were quite long, caused the VLM to hallucinate, resulting in far too many positive classifications (i.e., false detections of flooding). Further, all four more complex prompts, due to their length, were significantly slower to run on our dataset. For all these reasons, we used the prompt "Does the street in this image show more than a foot of standing water?" in our analysis. However, model performance and optimal prompting strategies are a continually evolving frontier, and we view experimenting with additional prompts and prompting strategies as an intriguing direction for future work: for example, one could investigate automatic prompt optimization frameworks, like Genetic-Pareto (GEPA) [114].

C Additional Bayesian modeling details

C.1 Model priors

We place the following weakly informative priors on model parameters:

$$\begin{aligned}
\alpha &\sim \text{Normal}(-5, 2)\\
\beta &\sim \text{Normal}(0, 2)\\
\theta_{\hat{y}=1|y=1} &\sim \text{Logit-Normal}(0, 2)\\
\theta_{\hat{y}=1|y=0} &\sim \text{Logit-Normal}(0, 2)
\end{aligned}$$

The negative-centered prior on α reflects the prior belief that most locations are not flooded. We place an ordered vector constraint on θ to enforce the assumption that $\theta_{\hat{y}=1|y=1} > \theta_{\hat{y}=1|y=0}$.

Our model of $p(y)$ also includes an ICAR spatial component ϕ:

$$p(y = 1|C = c) = \text{logit}^{-1}(\alpha + X_c\beta + \phi \cdot \sigma_\phi)$$

ICAR models are commonly used to capture spatially correlated data. We implement the ICAR component, as is standard, by incrementing the log probability using the pairwise difference

formula [86]:

$$\sum_{i,j:A_{ij}=1,i<j} -\frac{1}{2}(\phi_i - \phi_j)^2$$

where A denotes the adjacency matrix for Census areas. We place a soft sum-to-zero constraint on ϕ. We place a $\text{Normal}_+(0, 1)$ prior on the standard deviation of the ICAR component, σ_ϕ.

C.2 Flooding features used in Bayesian model

As an input to our Bayesian model (i.e., X_c in the notation above), we use 9 flood features from the external datasets described in Supplementary Information §A.2: the number of 311 reports in a Census tract, the number of FloodNet sensors in a tract, the minimum elevation of the tract, the mean elevation of the tract, the density of catch basins in a tract, the number of clogged catch basin complaints in a tract, the average clogged catch basin complaint resolution time in a tract, and the fraction of the tract with shallow and deep flooding in New York City Department of Environmental Protection Stormwater maps. We log-transform all right-skewed features, and z-score all features, prior to using them as inputs to the model, as is standard [115].

C.3 Comparison of Bayesian model to baselines

As described in Methods §M.2, we show that our Bayesian approach improves predictions of where flooded images will occur on a held-out test set, relative to both simple heuristics (e.g., the fraction of images which are classified as positive by the VLM) and machine learning baselines. We describe these baselines below.

C.3.1 Heuristic baselines

We compare to five heuristic baselines:

- **Fraction of positive classifications**: i.e., the fraction of images in a Census tract which the VLM classifies as positive.
- **Any positive classifications**: 1 if the VLM classifies any images in a Census tract as positive, 0 otherwise.
- **Number of positive classifications**: the number of images in a tract the VLM classifies as positive.
- **Any positive ground-truth annotations**: 1 if there are any images in a Census tract with ground-truth annotations as positive, 0 otherwise.
- **Number of positive annotations**: the number of images in a tract with ground-truth positive annotations.

We do not use the fraction of positive ground-truth annotations as a baseline because most tracts have no ground-truth annotations at all and for them this baseline is not well-defined.

C.3.2 Supervised learning baselines

For the supervised learning baselines, we use the same set of flood-relevant features which are inputs to our Bayesian model (Supplementary Information §C.2) to predict (a) the fraction of positive classifications and (b) the number of positive ground-truth annotations. We treat the variable to be predicted as a hyperparameter and report the setting which yields the best performance. We report results from both a linear regression model and a random forest model.

C.3.3 Graph smoothing baselines

We compare to *graph Laplacian smoothing baselines*, which use the graph Laplacian $L \triangleq D - A$ (where D is the diagonal degree matrix, and A is the adjacency matrix) to iteratively smooth a graph [116, 117]. One iteration of the algorithm updates the value $\mathbf{x}$ at each node (in our case, a Census tract) using the following diffusion update: $\mathbf{x}_{\text{new}} = \mathbf{x}_{\text{old}} - \alpha L \mathbf{x}_{\text{old}}$, where α is a step size parameter. The hyperparameters are α, the number of smoothing iterations, and the initial value to be smoothed (fraction of positive classifications or number of positive ground-truth annotations); we report the hyperparameter configuration which maximizes performance.

C.3.4 Comparison of baseline and Bayesian model performance

As described in Methods §M.2, we compare the performance of our Bayesian model to that of baselines by partitioning the classified images into a train set (which we use to fit the Bayesian model and the baselines on the classifications) and a test set (which we use to assess out-of-sample performance). The inputs to the Bayesian model and the baselines are the number of images in each Census tract with a given ground-truth annotation (positive, negative, or unknown) and a given VLM classification (positive or negative), as well as the flood-risk features X_c; some baselines make use of only a subset of this information. Neither the Bayesian model nor the baselines make use of the raw images themselves.

We use multiple metrics to compare the performance of our Bayesian model to baselines. First, we assess the out-of-sample Pearson correlation with fraction of images in the tract which are classified flooded. For this task, the output from our Bayesian model that we use is $p(y = 1|C = c)$, since this captures the fraction of images in a Census tract which are flooded.

We also assess the AUC in predicting whether (1) a Census tract will have any classified flooded images and (2) a Census tract will have any ground-truth annotated flooded images. For both these tasks, the output from our Bayesian model that we use is $p(\text{at least one image in tract is flooded}|C = c)$, since the goal is to predict the existence of a single image (as opposed to the fraction of flooded images).

On all of these metrics, our Bayesian model outperforms all baselines (Supplementary Table S5).

D Alternate prompts from prior work

In this section we describe the four alternate prompts from prior work we compared to, all of which were outperformed by our preferred prompt.

Assessing Large Multimodal Models for Urban Floodwater Depth Estimation (Basic) [51]

We tailor Lyu et al. [51]'s prompt for relative floodwater depth estimation to our street flooding detection task, defining Level 1 flooding or higher as 'flooded'. The full prompt text is as follows:

```
Analyze and estimate the deepest floodwater level of the image.
The estimated floodwater level should be one of the following:
- Level 0: 0 cm,
- Level 1: 1-15 cm,
- Level 2: 16-40 cm,
- Level 3: 41-60 cm,
- Level 4: 61-100 cm.
Give the answer with brief and reasonable explanations.
If the floodwater level is Level 1 or higher, answer Yes. Otherwise, answer No.
```

Assessing Large Multimodal Models for Urban Floodwater Depth Estimation (Advanced) [51]

The advanced version of Lyu et al. [51]'s prompt for relative floodwater depth estimation gives the VLM more contextual cues to look for, including vehicle wheels and the knees and waists of pedestrians. We employ the same tailoring as the basic prompt, classifying Level 1 flooding or higher as 'flooded'. The full prompt text is as follows:

```
As an expert in assessing floodwater levels from images,
utilize your expertise and the provided guidelines
to evaluate the floodwater level for each image.
Considerations:
1. Account for distractions in images.
2. Employ vehicles (cars, SUVs, and taxis, excluding buses and trucks) and people
as primary references for assessing floodwater levels.
Utilize additional indicators (water lines, water color, light reflection, etc.)
when primary references are insufficient.
3. Floodwater levels are categorized as follows:
```

```
Level 0: No water presence on the road.
Level 1: Water depth up to 15 cm, does not submerge vehicle
chassis or covering half of the wheels, or does not reach ankles.
Level 2: Water depth between 16-40 cm, exceeding Level 1 but not submerging fully wheels,
below the vehicle's front license plate, exhaust pipes, or not reaching knees.
Level 3: Water depth between 41-60 cm, exceeding Level 2 but not reaching above
the car's headlights, door handle, or not mid-thigh of people.
Level 4: Water depth between 61-100 cm, exceeding Level 3 but not full submersion half
of vehicle windows, or the car's bonnet, or people's waist.
4. Determine the highest floodwater level referenced in the image and report it
as the image's floodwater level.
Procedure:
1. Reference Floodwater Level: List each reference's floodwater level with a brief explanation.
2. Image Floodwater Level: Identify the image's floodwater level.
3. Answer: If the floodwater level is Level 1 or higher, answer Yes. Otherwise, answer No.
Notes:
1. Ensure a comprehensive understanding of the guidelines before proceeding.
2. Follow the procedure strictly and answer concisely.
```

A Novel Multimodal Large Language Model-Based Approach for Urban Flood Detection Using Open-Access Closed Circuit Television in Bandung, Indonesia [49]

Yang et al. [49]'s prompt is the best-performing external prompt, achieving a $p(y = 1|\hat{y} = 1)$ of 0.504 and a $p(y = 1|\hat{y} = 0)$ of 0.008 (Supplementary Table S4).The full prompt text is as follows:

```
Based on this road CCTV image, analyze if there is a flood or not.
Use this JSON example format:
{
"flood": true,
"confidence": 0.95,
"reasoning": "The road is submerged in water, indicating a flood."
}
If there is a flood, set "flood" to true; otherwise, set it to false.
The "confidence" should be a
float between 0 and 1, indicating your confidence in the prediction.
The "reasoning" should be a short explanation of your decision.
```

FloodVision [50]

We adapt Liu et al. [50]'s prompt for street passability under flooded conditions to our flooding detection task, replacing the original location context with 'New York City', and clarifying dashcam images as the visual medium. We classify a location as 'flooded' if this prompt classifies it as 'not passable' by a sedan. The full prompt text is as follows:

```
You are an engineering vision assistant.
From a single New York City street-view dashcam image frame, output:
  • Estimated standing-water depth at the flooded area (cm, integer)
  • Passability for ordinary sedan
  • Short justification

**Visual anchors & canonical dimensions**
  Raised planter curb reveal = 45 cm (+/- 5 cm)
  White parking-stripe width = 10 cm
  Concrete wheel stop height = 13 cm

**Cue-based depth bands**
  • Wheel stop fully visible; stripes crisp -> < 5 cm
```

- Water pooling at wheel stop base; stripes still crisp -> 5-10 cm
- Water near top of wheel stop; stripes slightly faded -> 10-15 cm
- Stripes faint / curb clearly above water -> 15-20 cm
- Stripes barely visible, water ~5 cm below curb -> 20-25 cm
- Stripes gone, water nearly level with curb -> 25-30 cm
- Water lapping over curb top -> 30-40 cm
- Wheel stop submerged & curb hidden -> > 40 cm

Passability rules
  - Sedan: pass <= 15 cm; caution 15-30 cm; unsafe > 30 cm

Required reasoning steps
1. Inspect stripe visibility and curb waterline.
2. Select the matching depth band.
3. Assign the band midpoint as depth_cm_estimate.
4. Determine sedan & ambulance verdict using the rules.
5. Output JSON ONLY.

Output format
```
{
  "depth_cm_estimate": ,
  "depth_range": "",
  "passability": "",
  "justification": ""
}
```

E Supplemental Figures

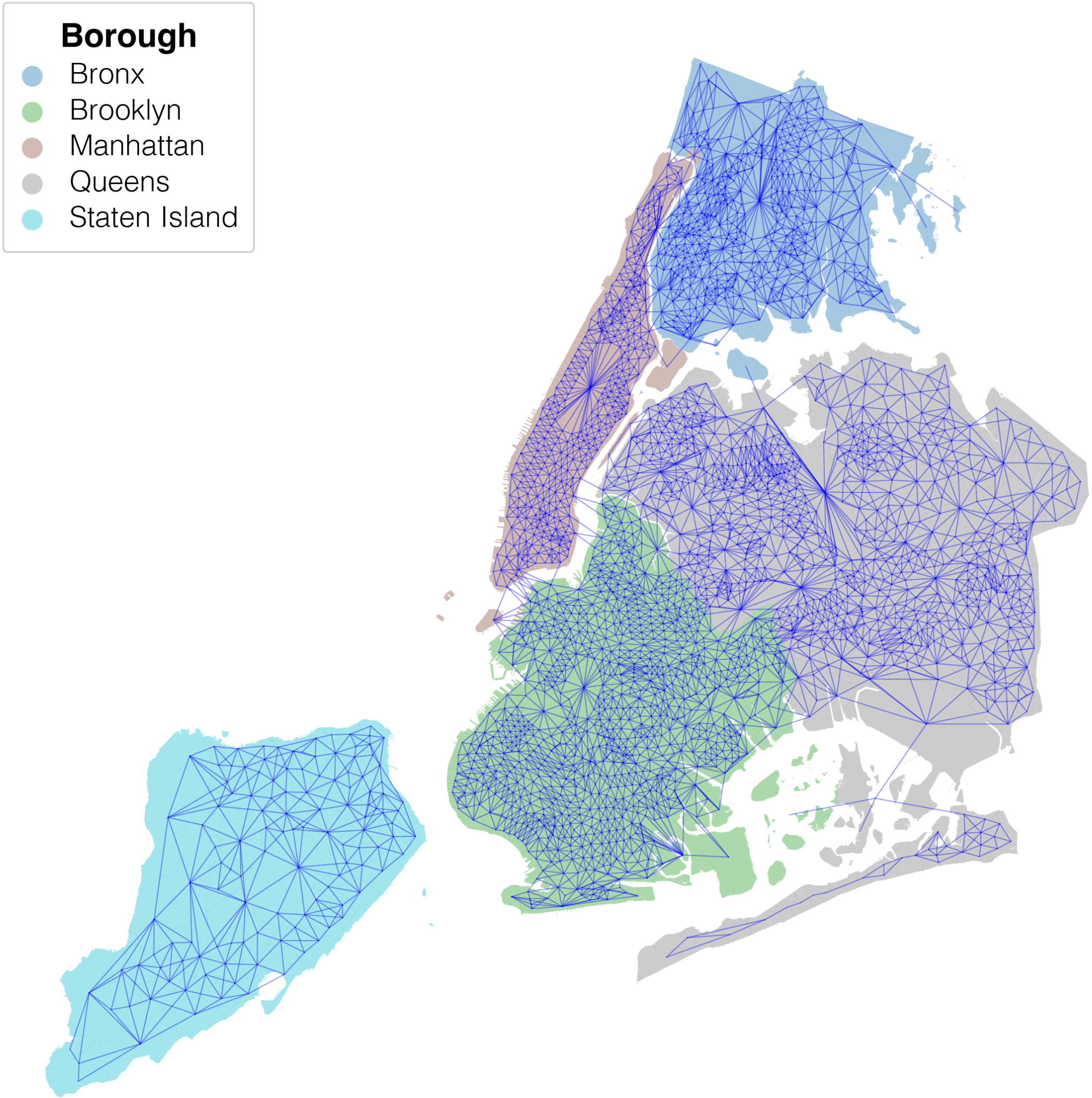

Fig. S1: Our adjacency network of NYC's Census tracts.

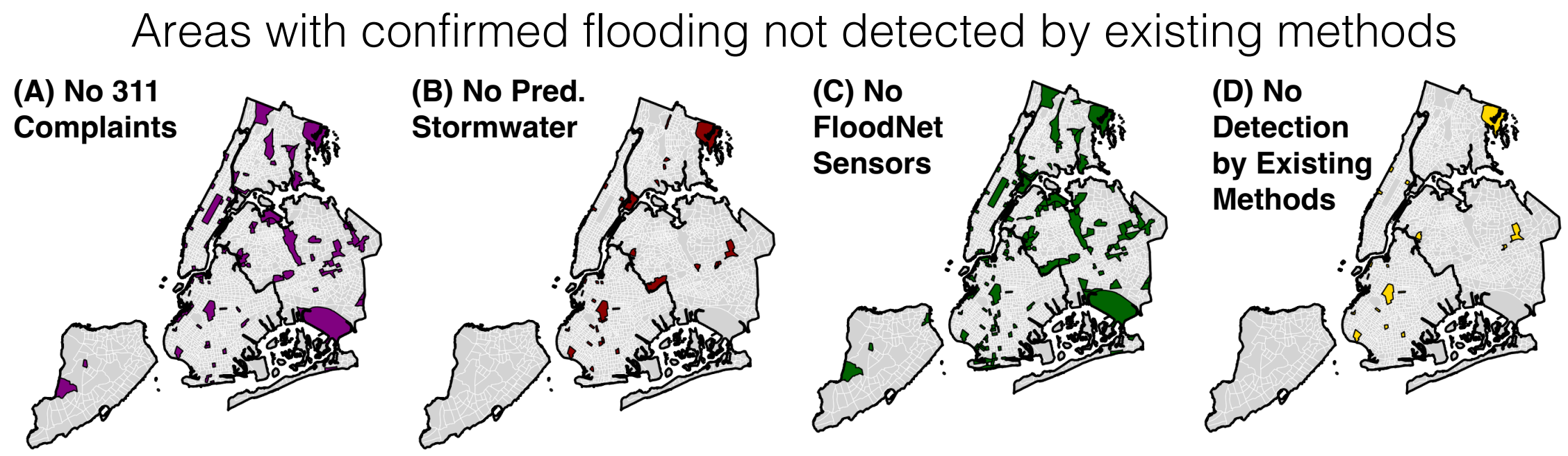

Fig. S2: We repeat the analysis in Figure 3A-D, but defining high-BayFlood-risk tracts as only those with a ground-truth annotated flooded image ($y = 1$). This approach identifies 45,003 residents living in areas with high BayFlood risk, but no coverage from existing flood detection methods, relative to 100,150 residents when incorporating the predictions of our Bayesian model, demonstrating the benefits of the Bayesian approach.

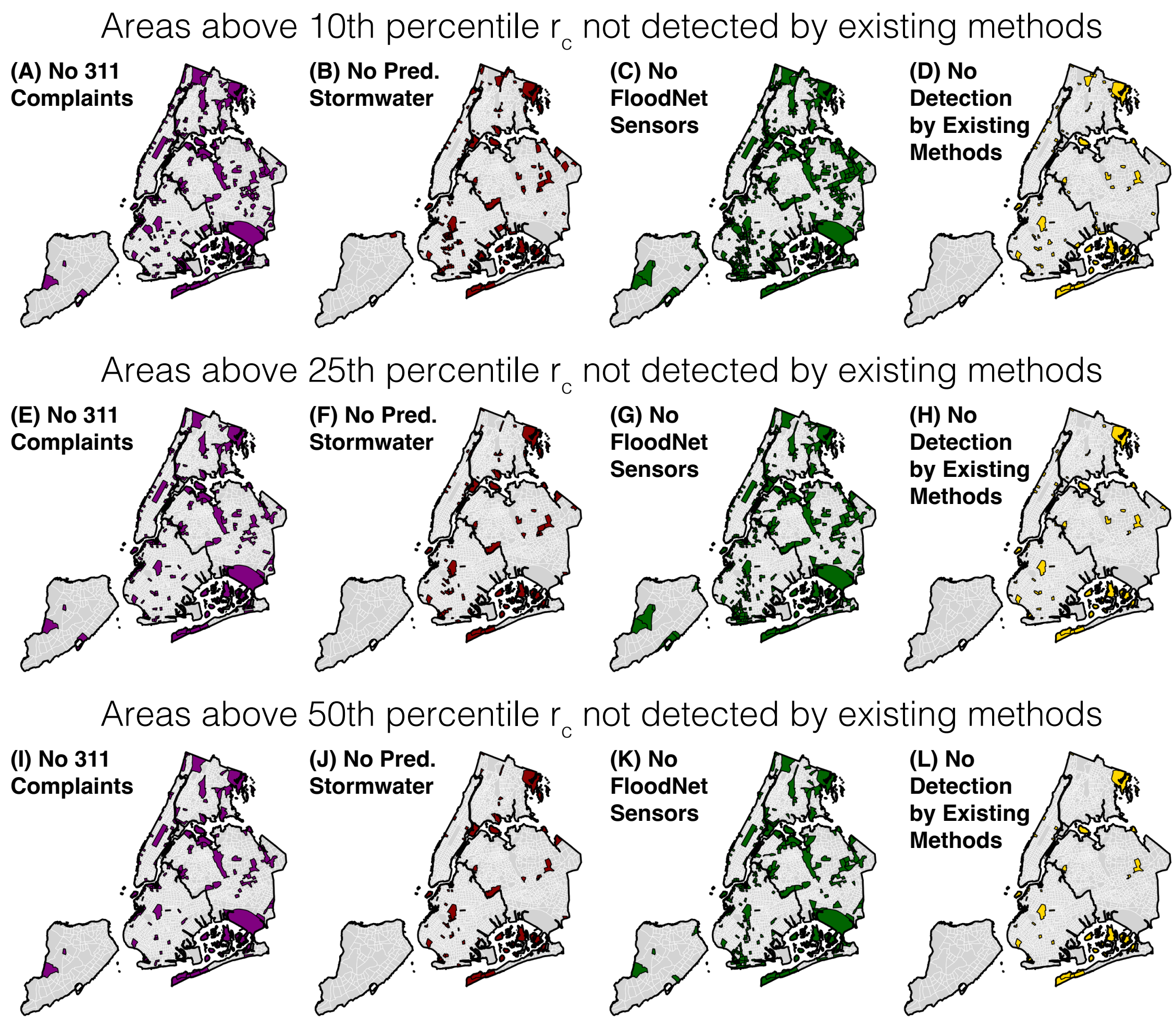

Fig. S3: We repeat the analysis in Figure 3A-D, but defining high-BayFlood-risk tracts using alternate thresholds for r_c: $r_c \geq$ 10th percentile among confirmed flooded images (top row of plots) and 50th percentile (bottom row of plots). For comparison, we also show the results from our primary analysis, with our original 25th percentile threshold, as the middle row of plots. The 10th percentile threshold identifies 198,091 residents living in areas with high BayFlood risk, but no coverage from existing flood detection methods, and the 50th percentile threshold identifies 60,683 residents (compared to 100,150 residents using a 25th percentile threshold, as in our main analysis).

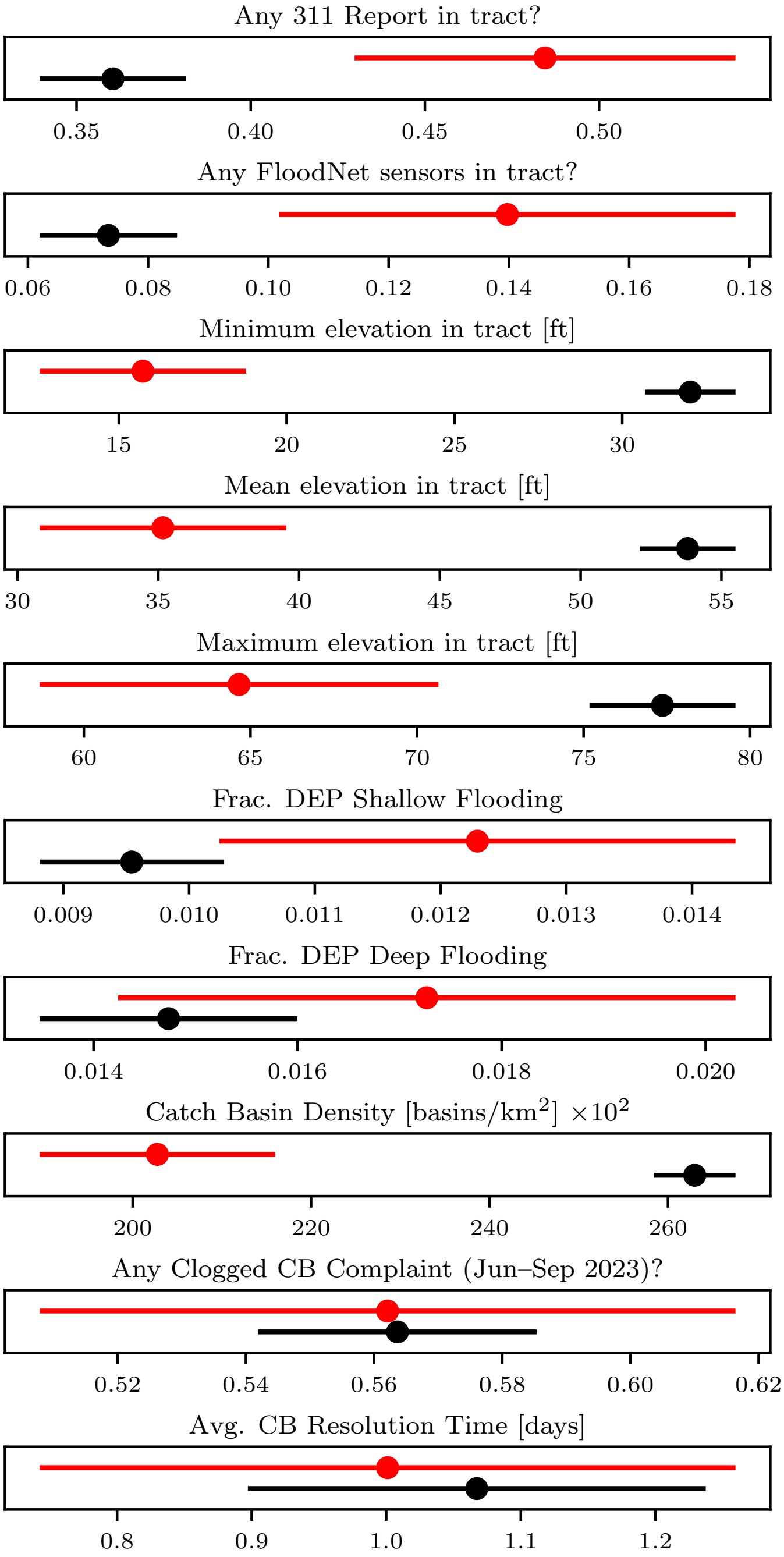

Fig. S4: Validation of BayFlood predictions against external flood-related features. "High BayFlood risk" refers to tracts c with either (1) $c \in C_{\text{confirmed}}$, where $C_{\text{confirmed}}$ is the set of all tracts with a confirmed ground-truth annotated flood image, or (2) $r_c > t$, where t is the 25th percentile of r_c among all tracts in $C_{\text{confirmed}}$. For this analysis only, we fit BayFlood without using any external flood-related features to allow validation of its predictions. All features except for any clogged catch basin complaint ($p = 0.96$), average clogged catch basin complaint resolution time ($p = 0.77$), and fraction of DEP 'deep' flooding ($p = 0.14$) are statistically significant at $\alpha = 0.05$, t-test. Error bars denote 95% confidence intervals.

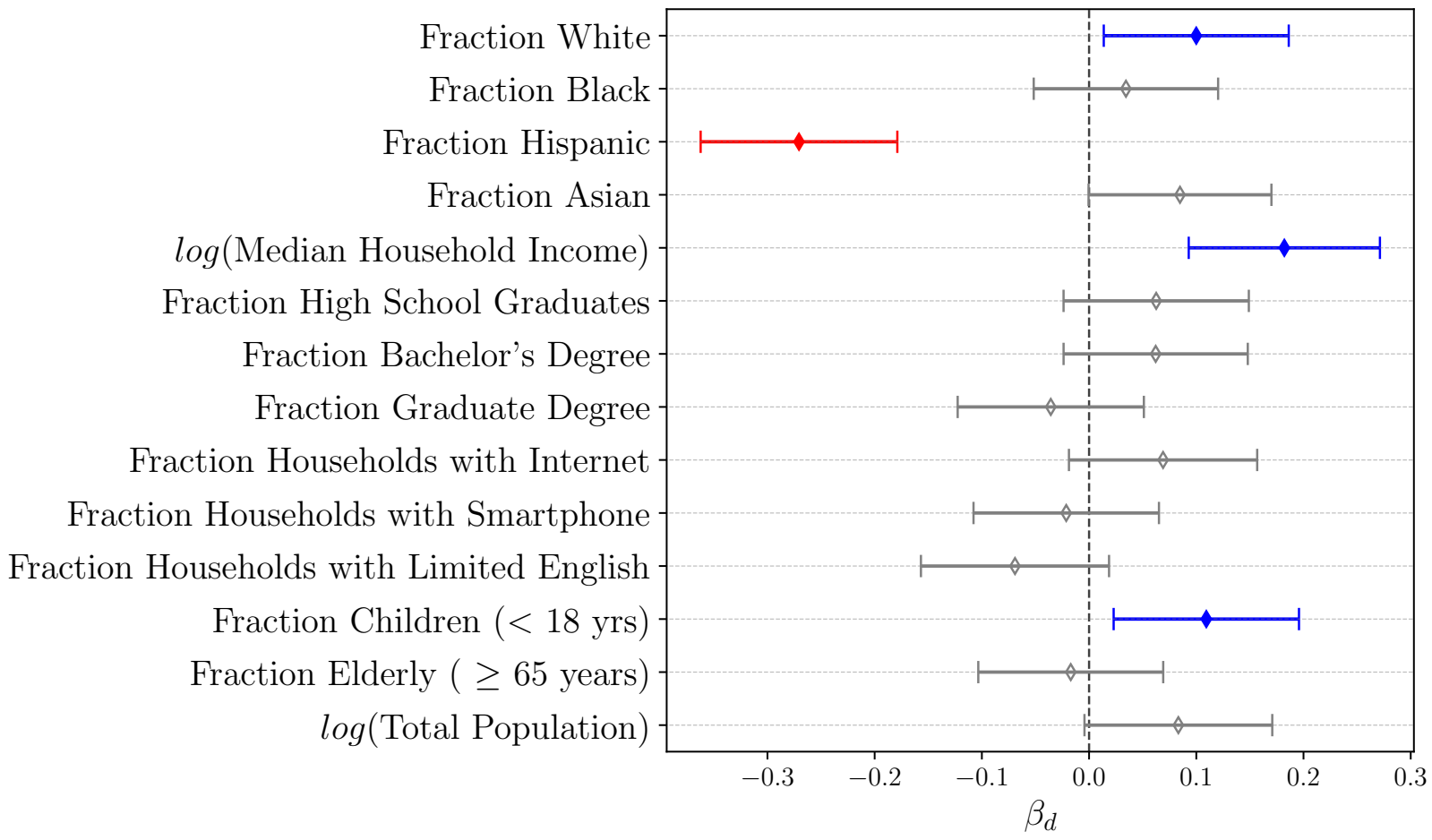

Fig. S5: Demographic coefficients for a risk-adjusted regression where we control for whether a tract has at least one confirmed flooded image as our measure of flood exposure (as opposed to controlling for r_c, as in our main results). Coefficients remain similar. Error bars denote 95% confidence intervals.

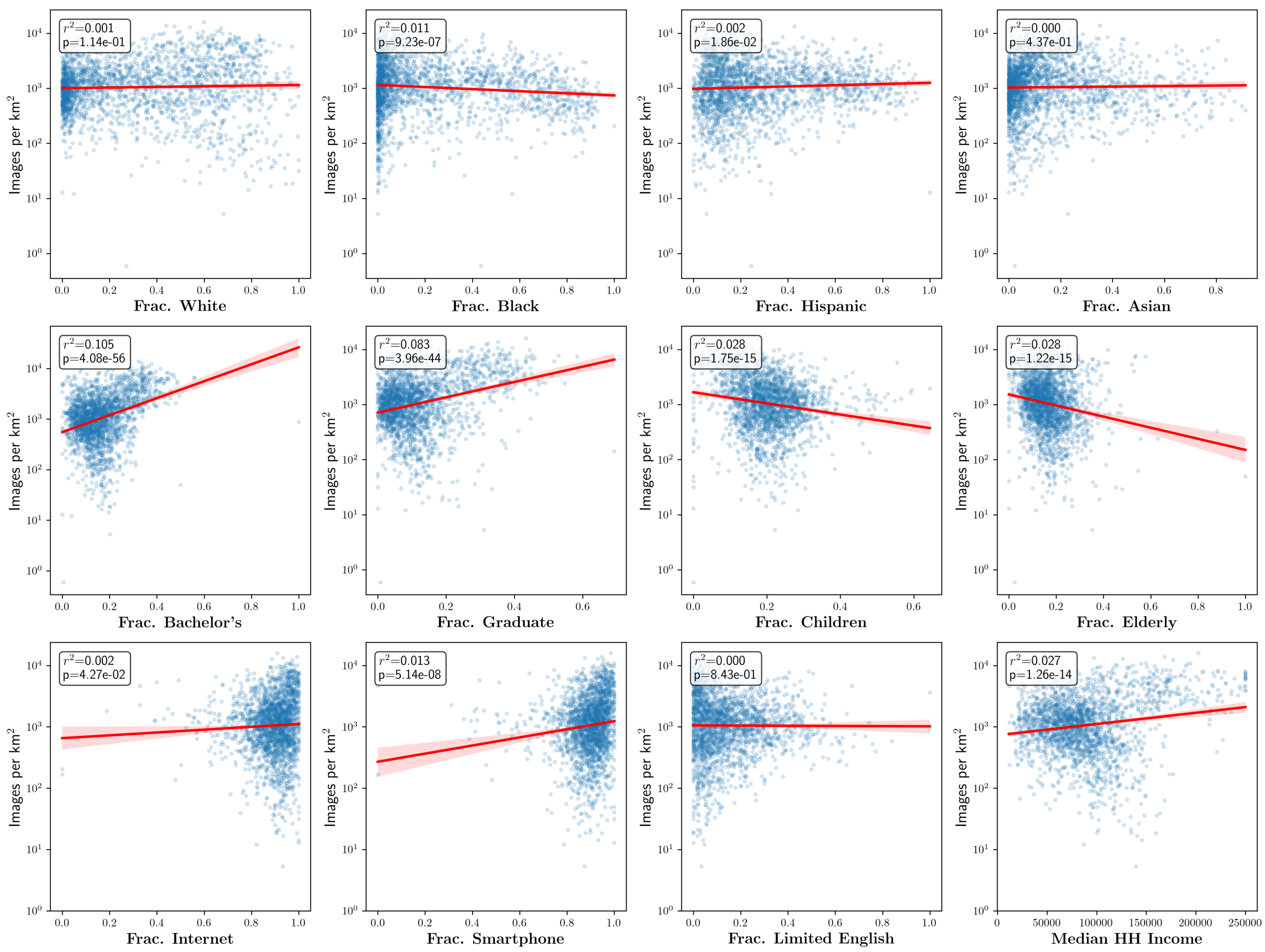

Fig. S6: Correlations between demographic variables and log-transformed dashcam image density across Census tracts. While some correlations are statistically significant, they are generally weak (maximum $R^2 < 0.11$).

F Supplemental Tables

Description	Data Source
Census and ACS Characteristics	
Total Population in Census tract	ACS DP05: Demographic and Housing Estimates [62]
Non-Hispanic White Population	ACS DP05: Demographic and Housing Estimates [62]
Non-Hispanic Black Population	ACS DP05: Demographic and Housing Estimates [62]
Hispanic Population	ACS DP05: Demographic and Housing Estimates [62]
Non-Hispanic Asian Population	ACS DP05: Demographic and Housing Estimates [62]
Number of Households with Internet Access	ACS 2801: Types of Computers and Internet Subscriptions [66]
Number of Households with Smartphone Access	ACS 2801: Types of Computers and Internet Subscriptions [66]
Median Annual Household Income (USD)	ACS S1901: Income in the Past 12 Months [65]
Number of High School Graduates	ACS S1501: Educational Attainment [63]
Number of Bachelor's Degree Holders	ACS S1501: Educational Attainment [63]
Number of Graduate Degree Holders	ACS S1501: Educational Attainment [63]
Number of Limited English Proficiency Households	ACS 1602: Limited English Speaking Households [64]
Physical Geography	
Minimum Elevation in Census tract (feet)	1 foot NYC Digital Elevation Model (DEM) [61]
Maximum Elevation in Census tract (feet)	1 foot NYC Digital Elevation Model (DEM) [61]
Mean Elevation in Census tract (feet)	1 foot NYC Digital Elevation Model (DEM) [61]
Geographic Area of Census tract (square feet)	2020 Census tracts Shapefile, NYC DCP [108]
Flood Infrastructure and Risk	
Number of Flooding-Related 311 Complaints	311 Service Requests, 2010-Present [13]
Number of FloodNet Sensors Installed	FloodNet Team (NYU, CUNY) [92]
Area of Shallow Flooding (4in–1ft) Under Moderate Rain (2.13in/hr)	NYC DEP Stormwater Maps [91]
Area of Deep Flooding (>1ft) Under Moderate Rain (2.13in/hr)	NYC DEP Stormwater Maps [91]
Distribution of Sewage Network Catch Basins	NYCDEP Citywide Catch Basins [60]
Number of Clogged Catch Basin Complaints	311 Service Requests, 2010-Present [13]
Average Clogged Catch Basin Complaint Resolution Time	311 Service Requests, 2010-Present [13]

Table S1: Fields from external datasets used in our analysis.

Date	Location	$p(y = 1 \| \hat{y} = 1)$	$p(y = 1 \| \hat{y} = 0)$
9/29/23	NYC	0.658 [0.616, 0.700]	0.006 [0.000, 0.014]
12/18/23	NYC	0.702 [0.606, 0.787]	0.000 [0.000, 0.000]
1/10/24	NYC	0.812 [0.625, 1.000]	0.000 [0.000, 0.000]
2/10/24	SF Bay	0.143 [0.000, 0.429]	0.000 [0.000, 0.000]

Table S2: Validation of VLM performance across multiple days and locations. We report the positive predictive value, $p(y = 1|\hat{y} = 1)$, and false omission rate, $p(y = 1|\hat{y} = 0)$. Classified positives ($\hat{y} = 1$) are much likelier to show flooding ($y = 1$) than classified negatives across all four days. Results reported are for our preferred model (Cambrian-1-13B); 95% bootstrap confidence intervals (100,000 samples) are provided in brackets.

Method	$p(y = 1 \mid \hat{y} = 1)$	$p(y = 1 \mid \hat{y} = 0)$
Supervised learning	0.464	0.012
CLIP	0.224	0.008
DeepSeek Janus-Pro-7B	0.248	0.012
Cambrian-1-8B	0.152	0.012
**Cambrian-1-13B (ours)**	**0.658**	**0.006**

Table S3: Comparison of our preferred VLM classifier (Cambrian-1-13B) to classification baselines on our primary dataset. Cambrian-1-13B achieves higher $p(y = 1|\hat{y} = 1)$ than all baselines ($p < 0.001$, t-test) and comparable $p(y = 1|\hat{y} = 0)$ to all baselines (differences not statistically significant).

Table S4: Comparison of our preferred prompt to five alternate prompts on our primary dataset. Our preferred prompt achieves higher $p(y = 1|\hat{y} = 1)$ than all alternate prompts ($p < 0.01$, t-test) and comparable $p(y = 1|\hat{y} = 0)$ to all alternate prompts (differences not statistically significant).

Prompt	$p(y = 1 \mid \hat{y} = 1)$	$p(y = 1 \mid \hat{y} = 0)$
**Ours (>1ft flooding)**	**0.658**	**0.006**
Ours (Basic Flooding Query)	0.540	0.004
Yang Advanced [49]	0.504	0.008
Lyu Advanced [51]	0.024	0.000
Liu FloodVision [50]	0.020	0.012
Lyu Basic [51]	0.000	0.000

Method	Pearson r [frac + classifications]	AUC [any ground-truth +]	AUC [any + classifications]
Frac. + classifications	0.55 ± 0.04	0.74 ± 0.01	0.67 ± 0.00
Any + classifications?	0.26 ± 0.01	0.74 ± 0.01	0.67 ± 0.00
# + classifications	0.30 ± 0.02	0.75 ± 0.01	0.68 ± 0.00
Any + annotations?	0.26 ± 0.02	0.62 ± 0.01	0.57 ± 0.00
# + annotations	0.27 ± 0.02	0.62 ± 0.01	0.57 ± 0.00
OLS	0.23 ± 0.01	0.78 ± 0.01	0.68 ± 0.00
Random Forest	0.56 ± 0.04	0.80 ± 0.01	0.72 ± 0.00
Laplacian smoothing	0.57 ± 0.04	0.81 ± 0.01	0.74 ± 0.00
**BayFlood**	$\mathbf{0.64 \pm 0.02}$	$\mathbf{0.89 \pm 0.00}$	$\mathbf{0.80 \pm 0.00}$

Table S5: Applying the Bayesian model to the VLM classifications improves out-of-sample prediction of three outcomes relative to baselines. From left to right, the columns report Pearson correlation with fraction of images classified positive; AUC for predicting whether there are any ground-truth positive annotations; and AUC for whether there are any positive VLM classifications. We report the mean and 95% confidence interval across 20 random train/test splits.

Supplementary Information References

[93] Kim, J., Jang, K.M.: An examination of the spatial coverage and temporal variability of Google Street View (GSV) images in small- and medium-sized cities: A people-based approach. Computers, Environment and Urban Systems **102**, 101956 (2023) https://doi.org/10.1016/j.compenvurbsys.2023.101956 . Accessed 2025-01-24

[94] Dadashova, B., Dobrovolny, C.S., Tabesh, M.: Detecting pavement distresses using crowd-sourced dashcam camera images. Technical report, Safety through Disruption (Safe-D) University Transportation Center (UTC) (2021). https://rosap.ntl.bts.gov/view/dot/60311 Accessed 2025-02-02

[95] Chowdhury, T., Mandel, I., Ortiz, J., Ju, W.: Designing a User-centric Framework for Information Quality Ranking of Large-scale Street View Images. arXiv. arXiv:2404.00392 [cs] (2024). https://doi.org/10.48550/arXiv.2404.00392 . http://arxiv.org/abs/2404.00392 Accessed 2025-02-02

[96] Chowdhury, T., Bhatti, A., Mandel, I., Ehsan, T., Ju, W., Ortiz, J.: Towards sensing urban-scale COVID-19 policy compliance in new york city. In: Proceedings of the 8th ACM International Conference on Systems for Energy-Efficient Buildings, Cities, And Transportation, pp. 353–356. ACM, Coimbra Portugal (2021). https://doi.org/10.1145/3486611.3491123 . https://dl.acm.org/doi/10.1145/3486611.3491123 Accessed 2025-02-02

[97] White, A., Trump, K.-S.: The Promises and Pitfalls of 311 Data. Urban Affairs Review **54**(4), 794–823 (2018) https://doi.org/10.1177/1078087416673202 . Accessed 2025-02-02

[98] Clark, B.Y., Brudney, J.L., Jang, S.-G.: Coproduction of government services and the new information technology: Investigating the distributional biases. Public Administration Review **73**(5), 687–701 (2013)

[99] O'Brien, D.T., Sampson, R.J., Winship, C.: Ecometrics in the age of big data: Measuring and assessing "broken windows" using large-scale administrative records. Sociological Methodology **45**(1), 101–147 (2015)

[100] Silverman, A.I., Brain, T., Branco, B., Challagonda, P.s.v., Choi, P., Fischman, R., Graziano, K., Hénaff, E., Mydlarz, C., Rothman, P., Toledo-Crow, R.: Making waves: Uses of real-time, hyperlocal flood sensor data for emergency management, resiliency planning, and flood impact mitigation. Water Research **220**, 118648 (2022) https://doi.org/10.1016/j.watres.2022.118648 . Accessed 2025-02-02

[101] FloodNet NYC: Community Engagement (2025). https://www.floodnet.nyc/home-1-1 Accessed 2025-02-02

[102] Van Alphen, J., Martini, F., Loat, R., Slomp, R., Passchier, R.: Flood risk mapping in Europe, experiences and best practices. Journal of Flood Risk Management **2**(4), 285–292 (2009) https://doi.org/10.1111/j.1753-318X.2009.01045.x . Accessed 2025-01-27

[103] Faber, J.W.: Superstorm Sandy and the demographics of flood risk in New York City. Human Ecology **43**, 363–378 (2015). Accessed 2025-01-27

[104] Rosenzweig, B., Montalto, F.A., Orton, P., Kaatz, J., Maher, N., Kleyman, J., Chen, Z., Sanderson, E., Adhikari, N., McPhearson, T., Herreros-Cantis, P.: NPCC4: Climate change and New York City's flood risk. Annals of the New York Academy of Sciences **1539**(1), 127–184 (2024) https://doi.org/10.1111/nyas.15175 . _eprint: https://onlinelibrary.wiley.com/doi/pdf/10.1111/nyas.15175. Accessed 2025-02-02

[105] NYC OpenData: NYC Stormwater Flood Maps (2024). https://experience.arcgis.com/experience/6f5482ced986448a8ba86a0e9bb8ad88/page/Page-1?views=Moderate-2.13-2%2CModerate-2.13-4 Accessed 2026-01-14

[106] NYC Mayor's Office of Resiliency: New York City Stormwater Resiliency Plan. Technical report (May 2021). https://www.nyc.gov/assets/orr/pdf/publications/stormwater-resiliency-plan.pdf Accessed 2026-01-14

[107] Record rain in NYC generates 'life-threatening' flooding, overwhelming streets and subways. ABC7 San Francisco (2023). Chap. weather. Accessed 2025-12-23

[108] NYC DCP: Census - Download and Metadata (2025). https://www.nyc.gov/site/planning/data-maps/open-data/census-download-metadata.page Accessed 2025-02-02

[109] Bertsch, R., Glenis, V., Kilsby, C.: Building level flood exposure analysis using a hydrodynamic model. Environmental Modelling & Software **156**, 105490 (2022)

[110] Lee, J.-H., Lee, S., Kim, B., Choi, H., Noh, S.J.: Evaluating the effects of spatial resolution on 2d pluvial flood modeling in urban built environments. Journal of Flood Risk Management **18**(3), 70105 (2025)

[111] Landwehr, T., Dasgupta, A., Waske, B.: Towards robust validation strategies for eo flood maps. Remote Sensing of Environment **315**, 114439 (2024)

[112] Altman, D.G., Bland, J.M.: Statistics notes: Diagnostic tests 2: predictive values. Bmj **309**(6947), 102 (1994)

[113] Chen, X., Wu, Z., Liu, X., Pan, Z., Liu, W., Xie, Z., Yu, X., Ruan, C.: Janus-Pro: Unified Multimodal Understanding and Generation with Data and Model Scaling. arXiv. arXiv:2501.17811 [cs] (2025). https://doi.org/10.48550/arXiv.2501.17811 . http://arxiv.org/abs/2501.17811 Accessed 2025-02-03

[114] Agrawal, L.A., Tan, S., Soylu, D., Ziems, N., Khare, R., Opsahl-Ong, K., Singhvi, A., Shandilya, H., Ryan, M.J., Jiang, M., Potts, C., Sen, K., Dimakis, A.G., Stoica, I., Klein, D., Zaharia, M., Khattab, O.: GEPA: Reflective Prompt Evolution Can Outperform Reinforcement Learning. arXiv. arXiv:2507.19457 [cs] (2025). https://doi.org/10.48550/arXiv.2507.19457 . http://arxiv.org/abs/2507.19457 Accessed 2025-12-23

[115] Friedman, J.: The elements of statistical learning: Data mining, inference, and prediction (2009). Accessed 2025-02-10

[116] Li, Q., Han, Z., Wu, X.-M.: Deeper insights into graph convolutional networks for semi-supervised learning. In: Proceedings of the AAAI Conference on Artificial Intelligence, vol. 32 (2018). https://ojs.aaai.org/index.php/AAAI/article/view/11604 Accessed 2025-02-10

[117] Chen, F.-C., Subedi, A., Jahanshahi, M.R., Johnson, D.R., Delp, E.J.: Deep Learning–Based Building Attribute Estimation from Google Street View Images for Flood Risk Assessment Using Feature Fusion and Task Relation Encoding. Journal of Computing in Civil Engineering **36**(6), 04022031 (2022) https://doi.org/10.1061/(ASCE)CP.1943-5487.0001025 . Accessed 2025-07-03
