# Supplementary material for: Improving flood detection with large-scale dashboard camera data
Source: Nat Commun. 2026 Jul 29;17:9208. doi: 10.1038/s41467-026-75938-1 (PMC13522533; doi:10.1038/s41467-026-75938-1)
Supplement: Supplementary file 2 [file 41467_2026_75938_MOESM2_ESM.pdf]

nature portfolio

Peer Review File

Improving flood detection with large-scale dashboard camera data

Corresponding Author: Professor Emma Pierson

This file contains all reviewer reports in order by version, followed by all author rebuttals in order by version.

Version 0:

Reviewer comments:

Reviewer #1

(Remarks to the Author)
This study presents very interesting results on the use of vision language models (VLMs) to identify urban flooded streets from ubiquitous dashcam footage, using an extreme rain event on September 29, 2023 in New York City as a case study. This approach is novel and will help to provide critically needed information on the extent of flooding in response to extreme events, since existing methods are inadequate. The paper is well-written and the authors provide access to their code and sufficient information about their data sources to support reproducibility. However, I have several suggestions to prevent the misinterpretation of the study results and to correct minor issues:

The most significant issue is the use of the term “flood risk”, which will be misinterpreted by many readers as applying across all rainfall conditions. In this study, the authors identify flooded areas during one event on September 29, 2023. The spatial extent of rainfall during this event was highly variable across the city, with the most intense rain occurring over western Brooklyn (e.g. Downtown, Gowanus, Carroll Gardens Prospect Heights, Park Slope) and, to a lesser extent, East New York and eastern Queens near JFK airport. This type of spatial variability is common during flood events in this region and, unsurprisingly, the worst flooding was observed in the areas that experienced the most intense rainfall. The authors should discuss the fact that rainfall spatial variability played a significant role in determining which areas experienced flooding during this event. Other areas of the city may generally be more at risk of inundation when they experience extreme rain, it just did not rain as much there during this particular event.

The authors should change their description of these areas to emphasize that the model only predicts their risk of flooding during this particular rain event. It would be more appropriate to use “flooded areas during this event” rather than just “flood risk”. For example:
- Lines 292-293 “Census-tract specific estimates of flood risk correlate with the previously mentioned external markers of flood risk” could be corrected to “Census-tract specific estimates of flooded areas correlate with the previously mentioned external markers of likely flooded areas”

If/where the term “flood risk” does continue to be used, it should at least be extended to “flood risk during this event”, to make clear that the risk is specifically associated with this rain event and may not be generally applicable across different storms.

Lines 157 and 161 “Hurricane Ida”: Ida was no longer a hurricane when it caused flooding in the New York City metropolitan area on September 1, 2021, and its remnants were only one of several meteorological factors that contributed to extreme rainfall that evening. This should be corrected to “Post-Tropical Cyclone Ida”

Line 692 Census and American Community [Survey] Datasets ("Survey" is missing here)

Lines 1204-1212: It would be really helpful for the reading to explain which of the 3 NYC DEP scenarios are used here. This information is already provided in Supplemental Table E, but it is essential information for the reader to interpret the use of the DEP data in context, so the use of scenarios should be briefly described here as well.

-BR Rosenzweig

(Remarks on code availability)
It would not be possible to run the model without access to the proprietary NEXAR dashcam data. However, the code is very well-documented and provides a usable resource for the community.

Reviewer #2

(Remarks to the Author)
The use of dashcam data for flood monitoring is an interesting idea, as it introduces a new data source for flood detection and addresses limitations of existing methods.The idea is of significance to the field, but I have some major concerns:

1. Currently, the study focuses solely on a “binary classification task” (identifying the presence/absence of flooding). Notably, recent research has demonstrated that visual models (e.g., YOLO, GPT, LLaVA) can estimate floodwater depth—for instance, by leveraging the relative height of water against car tires[1] or traffic signage[2]. Integrating floodwater depth estimation into the current framework, and further comparing its accuracy with other monitoring methods (e.g., flood sensors), would significantly elevate the study’s innovation and real-world applicability.

2. In Section 2.4, the analysis doesn’t account for urban drainage network systems when evaluating overlooked high-flood-risk areas and determining new flood sensor placement. drainage infrastructure directly influences flood occurrence and severity (e.g., outdated pipelines increase local flooding risk). Omitting this factor may compromise the accuracy of flood risk assessments and the rationality of sensor placement recommendations.

3. The prompt used for VLM classification “Does this image show more than a foot of standing water?” is overly simplistic. Additionally, the alternative prompts compared in Section B.3 remain too simplistic. Testing more multi-step or complex prompts and comparing their performance would help optimize the VLM’s classification accuracy.

Below are some specific recommendations:

4. In the Introduction, the literature review on “visual models in flood monitoring” should be expanded.

5. Lines 140–163 (content related to the dataset) are more appropriate for the **Methods section** rather than the Results. Relocating this content would improve the manuscript’s structure.

6. Lines 273–276: To derive the conclusion that “if the VLM predicts an image is flooded, it is xx more likely to be flooded,” the correct comparison should be between $p(y = 1 \mid \hat{y} = 1)$ and $p(y = 0 \mid \hat{y} = 1)$. The current comparison between $p(y = 1 \mid \hat{y} = 1)$ and $p(y = 1 \mid \hat{y} = 0)$ is methodologically inaccurate and may mislead readers about the VLM’s discriminative power.

7. Lines 282–290: The three validation datasets (for additional flood events in New York City and San Francisco) report no false negatives ($p(y = 1 \mid \hat{y} = 0)$), whereas the primary dataset (September 29, 2023, New York City flood) includes such errors. The manuscript should: (1) explain why false negatives are absent in the validation datasets; (2) characterize the image features of misclassified samples (e.g., ambiguous water reflections, occluded street views) in the primary dataset to clarify the VLM’s limitations.

8. Lines 298–300: The rationale for using the 25th percentile to define “high BayFlood risk” is unclear. The manuscript should justify this threshold.

9. Lines 325–328 and Lines 297–300 convey nearly identical content (both defining “high BayFlood risk” tracts). This redundancy should be eliminated to streamline the text.

10. Lines 345–348 & Lines 395–397 (details on the risk-adjusted logistic regression for 311 reporting biases) belong in the “Methods section” (as they describe analytical procedures). Additionally: (1) the manuscript does not report the statistical significance of regression coefficients; (2) it is unclear whether the demographic variable was included in the regression “one at a time” (univariate regression) or “simultaneously” (multivariate regression).

11. Lines 428–432 and Lines 444–446 (in the Discussion) contain redundant descriptions of the study’s application in sensor placement. One of these sections should be removed or condensed.

12. Line 553–554: The annotation criterion states that an image is “annotated as positive if it showed definite flooding: namely, the street in front of the vehicle was visible and showed significant flooding.” This is counterintuitive—flooding typically obscures street details, so it is unclear why a “visible street” is used as a criterion for labeling flooding.

References:
[1] Lyu, H., Zhou, S., Wang, Z., Fu, G., & Zhang, C. (2025). Assessing large multimodal models for urban floodwater depth estimation. Water Resources Research, 61, e2024WR039494. https:// doi.org/10.1029/2024WR039494
[2] Alizadeh, B., & Behzadan, A. H. (2023). Scalable flood inundation mapping using deep convolutional networks and traffic

signage. Computational Urban Science, 3(1), 17. https://doi.org/10.1007/s43762-023-00090-1

(Remarks on code availability)

Reviewer #3

(Remarks to the Author)
This manuscript presents an innovative approach to urban flood detection by leveraging large-scale dashboard camera (dashcam) datasets combined with vision–language models (VLMs). The study introduces BAYFLOOD, a two-stage framework that first applies zero-shot classification to identify flooded street scenes and then uses Bayesian spatial modeling for prediction refinement. The work addresses major limitations in current flood detection methods (311 reports, FloodNet sensors, and modeled stormwater accumulation with elevation data), particularly the issues of poor spatiotemporal granularity and inequitable coverage. Key contributions include demonstrating improved flood detection accuracy in New York City, quantifying demographic biases in 311 reporting systems, and providing actionable recommendations for optimal flood sensor placement.

This is a high-quality and technically sound manuscript that makes meaningful methodological contributions toward the utilization of large-scale dashcam imagery for environmental monitoring. The study addresses an important societal challenge in urban resilience and data equity. However, there are several substantial concerns regarding the authors' understanding of hydrologic processes and the robustness of their validation methods. I recommend acceptance after major revisions, addressing the following key points—particularly the concerns on annotation reliability and spatial granularity.

1. As described in lines 551–555, the manual annotation process relied on a single individual labeling 1,000 ground-truth images to ensure "consistency." While consistency across annotators is valuable, the subjective nature of judging "definite flooding" versus "small puddles" raises significant concerns regarding data reliability. In flood modeling, water depth is a critical parameter in defining inundation, and visual ambiguity can easily affect classification outcomes.
It is strongly recommended to (a) assess inter-annotator agreement using a subset of images labeled by multiple annotators, or (b) explicitly acknowledge the limitation of using a single annotator and clearly define the operational threshold for what constitutes "flooding." This would enhance transparency and reproducibility.

2. While the paper emphasizes the fine-grained nature of dashcam data compared to sensors or static maps (line 27), the final analysis is aggregated to the Census Tract level, which remains relatively coarse. The claim that 95% of tracts contain at least 50 images also implies that 5% of tracts have fewer samples, raising questions about data representativeness during flood events.
Please justify:
- Why the Census Tract level was selected instead of more granular units such as Census Blocks or Block Groups.
- Whether the number of images per tract is sufficient to capture spatial variability in flooding within those tracts.
- How the Bayesian spatial model compensates for sparse data, particularly in tracts with low image counts, and whether this compensation is validated quantitatively.

3. It is unclear what the temporal and spatial coverage of the data is, and the methodology appears to be constrained by its data source. Dashcams inherently provide only street-level perspectives from moving vehicles. I recommend that the authors explicitly discuss two key limitations in the Discussion section:
(a) The method cannot capture inundation occurring away from the roadway (e.g., in parks, private properties, or beneath structures such as bridges that are not visible from the street).
(b) The method is subject to selection bias during severe flood events, as areas that become too dangerous or inaccessible (e.g., due to road closures) are unlikely to be recorded by dashcam-equipped vehicles. This leads to blind spots precisely where flood risks are highest, meaning the risk estimates reflect only observable incidents rather than the full spatial extent of flood occurrence.
In addition, there is no analysis or discussion of how the vision–language model's (VLM) classification performance varies by time of day or lighting conditions. Because flooding can occur at any hour, image degradation at night—caused by low illumination, reflections, or glare—can substantially affect detection accuracy. I recommend evaluating the model's robustness under nighttime conditions. If possible, the authors should include a validation comparing performance between day and night images; otherwise, they should clearly state that the analysis is limited to daytime imagery due to the challenges of low-light environments.

4. The authors state that the proposed approach detected flooded areas missed by the existing static predictive map (NYC DEP stormwater map). However, comparing real-world flooding observations from September 29, 2023, with a static map designed for a specific rainfall magnitude (e.g., a 100-year event) and limited to large, contiguous areas (>0.25 acres) may mislead readers. The two datasets represent fundamentally different temporal and event-based contexts, and this distinction should be clarified. In addition, the reference for the NYC stormwater flood map appears to be incorrect. The manuscript cites reference [52], which does not contain the underlying data source. The correct source seems to be the NYC Open Data portal: https://data.cityofnewyork.us/Environment/NYC-Stormwater-Flood-Maps/9i7c-xyvv/about_data. Finally, I recommend revising the Introduction or Related Work sections to include recent advancements in high-resolution and physics-based urban flood modeling, which provide more accurate predictive capabilities. Notable studies include Guo et al. (2020), Schubert et al. (2022), Wing et al. (2017), and Noh et al. (2019).

5. The authors employ specialized metrics for evaluating the VLM (Positive Predictive Value and False Omission Rate). While these choices are reasonable given the highly imbalanced dataset (0.2% positive cases), the manuscript omits several conventional classification metrics commonly reported for completeness—such as F1-score, Recall, and the Critical Success Index (CSI), which are frequently used in urban flooding studies. The absence of these metrics or a clear rationale for their exclusion limits the interpretability of the reported performance. I recommend that the authors either (a) provide a more comprehensive set of evaluation metrics for the VLM classification (e.g., in Supplementary Table S2), or (b) include a detailed justification explaining why PPV and FOR were considered the most appropriate and sufficient measures for this highly imbalanced, safety-critical detection context (see Bertsch et al., 2022; Lee et al., 2025).

6. The core comparison in this study involves relating point-based dashcam detections to polygon-based flood maps. However, the authors do not report any metric to evaluate spatial false negatives—that is, areas identified as flooded on the reference map but not captured by dashcam observations. The absence of such a spatial recall assessment limits the evaluation of the method's completeness and reliability. It is recommended that the authors address this limitation in the Discussion section. They should explicitly acknowledge the methodological challenge of comparing point observations to areal data and, if possible, implement a simple spatial performance measure—such as calculating the proportion of flood map polygons (or total inundated area) with no corresponding dashcam detections. This addition would substantially strengthen the credibility and interpretability of the results.

7. Line 59–62: The cited references [17–19] do not appear to substantiate the statement that "these forecasts can be imperfect, particularly as climate change alters historical weather patterns, diminishing their reliability and effectiveness in predicting future flood scenarios." Please revise this section to ensure that the references directly support the claim or provide additional citations that do so.

8. The manuscript does not clearly explain why the validation for the San Francisco dataset yields a substantially lower Positive Predictive Value (0.143) compared to New York City. This raises concerns about the consistency and generalizability of the proposed method across different geographic contexts, rainfall intensities, and event characteristics. The authors should clarify potential causes of this discrepancy and discuss the implications for the model's robustness and transferability.

References used:

Guo, K., Guan, M., & Yu, D. (2020). Urban surface water flood modelling–a comprehensive review of current models and future challenges. Hydrology and Earth System Sciences Discussions, 2020, 1-27.

Schubert, J. E., Luke, A., AghaKouchak, A., & Sanders, B. F. (2022). A framework for mechanistic flood inundation forecasting at the metropolitan scale. Water Resources Research, 58(10), e2021WR031279.

Wing, O. E., Bates, P. D., Sampson, C. C., Smith, A. M., Johnson, K. A., & Erickson, T. A. (2017). Validation of a 30 m resolution flood hazard model of the conterminous United States. Water Resources Research, 53(9), 7968-7986.

Noh, S. J., Lee, J. H., Lee, S., & Seo, D. J. (2019). Retrospective dynamic inundation mapping of Hurricane Harvey flooding in the Houston metropolitan area using high-resolution modeling and high-performance computing. Water, 11(3), 597.

Bertsch, R., Glenis, V., & Kilsby, C. (2022). Building level flood exposure analysis using a hydrodynamic model. Environmental Modelling & Software, 156, 105490.

Lee, J. H., Lee, S., Kim, B., Choi, H., & Noh, S. J. (2025). Evaluating the Effects of Spatial Resolution on 2D Pluvial Flood Modeling in Urban Built Environments. Journal of Flood Risk Management, 18(3), e70105.

(Remarks on code availability)

Version 1:

Reviewer comments:

Reviewer #1

(Remarks to the Author)
The authors have adequately addressed all of my previous comments in the revised manuscript.

BR Rosenzweig

(Remarks on code availability)
It would not be possible to run the model without access to the proprietary NEXAR dashcam data. However, the code is very well-documented and provides a usable resource for the community

Reviewer #2

(Remarks to the Author)
The manuscript has been improved substantially. All my concerns have been well addressed. I would like to recommend acceptance.

(Remarks on code availability)

Reviewer #3

(Remarks to the Author)
I appreciate the authors' effort in revising the manuscript. However, I still have several suggestions and questions:

1. The authors incorporated an inter-annotator agreement analysis following my previous suggestion. While the reported Cohen's kappa value (0.61) is interpreted as "substantial agreement," it can also be classified as "moderate" according to McHugh (2012). Given that this dataset serves as benchmark data, a more rigorous standard of agreement should be applied. Furthermore, the agreement rate for the "not flooded" class is relatively low (66%), which is concerning. This may stem from the lack of a clear quantitative threshold or rule to distinguish between "flooded" and "not flooded," suggesting that the benchmark labels may have been assigned subjectively.

2. The authors emphasize the fine-grained nature of dashcam data compared to other approaches; however, their response also indicates that the proposed method does not provide sufficient granularity at the census tract level. This limitation should be more explicitly acknowledged, particularly in terms of spatial coverage. While the data may be fine-grained at the image level, its spatiotemporal coverage appears limited.

Additionally, it would be valuable to evaluate whether image density is associated with socioeconomic characteristics (e.g., fewer images collected in underserved communities), as highlighted by Desouza & Kinney (2021).

Given that census tracts can span several square miles, a small number of images may not adequately capture within-tract variability. Although the Bayesian spatial model helps address data sparsity, it remains unclear whether the image density is sufficient to support analysis of urban flooding, which is highly dynamic and rapidly changing.

3. I agree that the F1 score is not appropriate for imbalanced datasets. However, the Critical Success Index (CSI), which excludes true negatives, provides a more informative measure of performance under class imbalance and should be considered.

Referece
McHugh, M. L. (2012). Interrater reliability: the kappa statistic. Biochemia medica, 22(3), 276-282.

Desouza, P., & Kinney, P. L. (2021). On the distribution of low-cost PM2. 5 sensors in the US: demographic and air quality associations. Journal of exposure science & environmental epidemiology, 31(3), 514-524.

(Remarks on code availability)

Version 2:

Reviewer comments:

Reviewer #3

(Remarks to the Author)
The authors have adequately addressed all of the comments I raised in my previous review.

(Remarks on code availability)

Open Access This Peer Review File is licensed under a Creative Commons Attribution 4.0 International License, which permits use, sharing, adaptation, distribution and reproduction in any medium or format, as long as you give appropriate credit to the original author(s) and the source, provide a link to the Creative Commons license, and indicate if changes were made.
In cases where reviewers are anonymous, credit should be given to 'Anonymous Referee' and the source.
The images or other third party material in this Peer Review File are included in the article's Creative Commons license, unless indicated otherwise in a credit line to the material. If material is not included in the article's Creative Commons license and your intended use is not permitted by statutory regulation or exceeds the permitted use, you will need to obtain permission directly from the copyright holder.
To view a copy of this license, visit https://creativecommons.org/licenses/by/4.0/

Emma Pierson
Assistant Professor
Department of Electrical Engineering and Computer Sciences
Computational Precision Health Program
emmapierson@berkeley.edu
https://people.eecs.berkeley.edu/ emmapierson/

We are grateful to the reviewers and editor for their meticulous assessment of the manuscript and their thoughtful and helpful feedback. We were glad to see that they found our approach provides "critically needed information on the extent of flooding in response to extreme events", and found the manuscript "novel", "innovative", "very interesting", "well-written", "high-quality" and "technically sound". We have comprehensively revised the manuscript and performed additional experiments to respond to all questions and concerns from the reviewers, and believe the manuscript to be significantly improved. We summarize the most important changes here:

- We have performed many additional experiments to strengthen the manuscript's empirical contribution and rigorously validate its claims. These include: (1) new analyses evaluating the ability of vision–language models to estimate relative flood depth near physical flood sensors, enabling direct comparison with ground-truth water-level measurements; (2) incorporation of stormwater drainage infrastructure data into our model, and as a validation; (3) analysis of additional and more complex prompts for flood detection; (4) calculation of new metrics characterizing model performance; and (5) additional robustness checks and sensitivity analyses across hyperparameters used in our analysis. Collectively, these experiments substantially strengthen the empirical foundation of the work and address key concerns raised by the reviewers regarding robustness, interpretability, and real-world applicability. Note that, because we have now incorporated additional data on stormwater drainage infrastructure to our model at the suggestion of the reviewers, our model predictions (and thus some numbers throughout the paper) have slightly changed, but conclusions remain very similar.
- We have significantly improved the clarity and precision of the manuscript by revising or removing ambiguous or confusing language; refining the spatial and event-specific framing; clarifying methodological details; and restructuring portions of the manuscript to improve clarity and flow.

We provide a point-by-point response to the reviewers' questions and suggestions below.

Reviewer 1

This study presents very interesting results on the use of vision language models (VLMs) to identify urban flooded streets from ubiquitous dashcam footage, using an extreme rain event on September 29, 2023 in New York City as a case study. This approach is novel and will help to provide critically needed information on the extent of flooding in response to extreme events, since existing methods are inadequate. The paper is well-written and the

authors provide access to their code and sufficient information about their data sources to support reproducibility.

Response: We thank Reviewer 1 for the complimentary feedback on our study, and are glad that they found the approach novel and helpful and the manuscript interesting, well-written, and reproducible.

However, I have several suggestions to prevent the misinterpretation of the study results and to correct minor issues:

The most significant issue is the use of the term "flood risk", which will be misinterpreted by many readers as applying across all rainfall conditions. In this study, the authors identify flooded areas during one event on September 29, 2023. The spatial extent of rainfall during this event was highly variable across the city, with the most intense rain occurring over western Brooklyn (e.g. Downtown, Gowanus, Carroll Gardens Prospect Heights, Park Slope) and, to a lesser extent, East New York and eastern Queens near JFK airport. This type of spatial variability is common during flood events in this region and, unsurprisingly, the worst flooding was observed in the areas that experienced the most intense rainfall. The authors should discuss the fact that rainfall spatial variability played a significant role in determining which areas experienced flooding during this event. Other areas of the city may generally be more at risk of inundation when they experience extreme rain, it just did not rain as much there during this particular event.

The authors should change their description of these areas to emphasize that the model only predicts their risk of flooding during this particular rain event. It would be more appropriate to use "flooded areas during this event" rather than just "flood risk". For example: "Lines 292-293 "Census-tract specific estimates of flood risk correlate with the previously mentioned external markers of flood risk" could be corrected to "Census-tract specific estimates of flooded areas correlate with the previously mentioned external markers of likely flooded areas". If/where the term "flood risk" does continue to be used, it should at least be extended to "flood risk during this event", to make clear that the risk is specifically associated with this rain event and may not be generally applicable across different storms.

Response: Thanks, this is a great point — we agree that the term "flood risk" could be misleading without further context. We have replaced the term "predicted flood risk" with "predicted flooded fraction" and similar language to emphasize that we are referring to predicted flooding only during this single rainfall event, not across events. We have also explicitly highlighted the point that rainfall spatial variability plays a significant role. Please let us know if you think further linguistic changes would help clarify.

Lines 157 and 161 "Hurricane Ida": Ida was no longer a hurricane when it caused flooding in the New York City metropolitan area on September 1, 2021, and its remnants were only one of several meteorological factors that

contributed to extreme rainfall that evening. This should be corrected to "Post-Tropical Cyclone Ida"

Response: Thanks very much for catching this! We have updated Section 2.1 to refer to the more accurate categorization of "Post-Tropical Cyclone Ida".

Line 692 Census and American Community [Survey] Datasets ("Survey" is missing here)

Response: Thanks — we have fixed this!

Lines 1204-1212: It would be really helpful for the reading to explain which of the 3 NYC DEP scenarios are used here. This information is already provided in Supplemental Table E, but it is essential information for the reader to interpret the use of the DEP data in context, so the use of scenarios should be briefly described here as well.

Response: We agree this would be useful to clarify! We now include an overview of the four scenarios, our selection, and rationale for our selection in Supplementary Information Section A.2.

It would not be possible to run the model without access to the proprietary NEXAR dashcam data. However, the code is very well-documented and provides a usable resource for the community.

Response: Thank you! We are glad you think the code is well-documented and hope that it provides a useful resource for the community. We would also love to be able to release the Nexar data, as we have done for the past projects; we are working hard on this but cannot commit to it at present.

Reviewer 2

The use of dashcam data for flood monitoring is an interesting idea, as it introduces a new data source for flood detection and addresses limitations of existing methods.

Response: We are glad you found the approach interesting and believed it could address limitations of current methods.

The idea is of significance to the field, but I have some major concerns: 1. Currently, the study focuses solely on a "binary classification task" (identifying the presence/absence of flooding). Notably, recent research has demonstrated that visual models (e.g., YOLO, GPT, LLaVA) can estimate floodwater depth—for instance, by leveraging the relative height of water against car tires[1] or traffic signage[2]. Integrating floodwater depth estimation into the current framework, and further comparing its accuracy with other monitoring methods (e.g., flood

sensors), would significantly elevate the study's innovation and real-world applicability.

Response: Thanks for this suggestion — we agree this is an interesting and important experiment to perform! We therefore investigated Cambrian-1-13B's capability for flood depth estimation, in line with recent similar work[1,2]. In order to validate this, we needed ground truth information on flood depth to which we could compare our model estimates. To obtain this ground truth, we filtered for all dashcam images within 15 meters of an active FloodNet sensor. Unfortunately, this resulted in only 107 images that exclusively occurred during periods of no flooding or shallow flooding, too small and imbalanced a dataset for reliable validation. We therefore decided to omit these results from the paper; however, we describe them here in case they are of interest, have also mentioned this in the Discussion as an important direction for future work, and are happy to include more of the details below in the manuscript if the reviewers and editor prefer!

We employed two prompts for relative flood depth estimation — one 'basic' prompt and one 'advanced' prompt, sourced from Lyu et al[1]. The 'basic' prompt solely contains a leveling scheme of Level 0-4, each corresponding to a higher range of standing floodwater (from 0 cm in Level 0 to 100 cm in Level 4), along with an instruction to provide a reasoning/explanation for the classification. The 'advanced' prompt supplements the basic prompt by adding contextual reference cues, including vehicle tires and pedestrians walking in the water, to guide the classification of each level; it also employs chain-of-thought reasoning via a two-step output procedure. We used each prompt to classify each of the 107 images into one of five levels with Cambrian-1-13B: Level 0 (0 cm of floodwater), Level 1 (1-15 cm), Level 2 (16-40 cm), Level 3 (41-60 cm), or Level 4 (61-100 cm). Both prompts allowed the VLM to return 'inconclusive' if a flooding determination was too difficult to make. We compared the VLM estimates to the ground truth depth estimates from the FloodNet sensor.

Of the 107 images, the vast majority corresponded to shallow flooding: 91% had a ground truth FloodNet depth estimate of Level 0; 9% of level 1; and none of level 2 or more. Overall, our sample size is too small, and the sample too imbalanced, to reliably validate depth estimation performance. However, we do observe some qualitatively interesting patterns. Firstly, the contextual cues and added detail of the advanced prompt produced better performance: at the image-level, 90% of Cambrian's predictions with the basic prompt are inconclusive, compared to 44% for the advanced prompt. Secondly, both prompts often *overestimated* floodwater depth, often due to hallucinations of relative depth cues. (It is unclear

[1] Heng Lyu et al. "Assessing Large Multimodal Models for Urban Floodwater Depth Estimation". en. In: *Water Resources Research* 61.4 (Apr. 2025), e2024WR039494

[2] Bahareh Alizadeh and Amir H. Behzadan. "Scalable flood inundation mapping using deep convolutional networks and traffic signage". en. In: *Computational Urban Science* 3.1 (Apr. 2023), p. 17

if this result would persist, however, in a dataset that was less skewed towards shallow or no flooding.) Third, we see fewer images taken during the *peak* of flooding as reported by a FloodNet sensor; this suggests that severe flooding may indeed reduce dashcam coverage, as we note in the Discussion.

2. In Section 2.4, the analysis doesn't account for urban drainage network systems when evaluating overlooked high-flood-risk areas and determining new flood sensor placement. drainage infrastructure directly influences flood occurrence and severity (e.g., outdated pipelines increase local flooding risk). Omitting this factor may compromise the accuracy of flood risk assessments and the rationality of sensor placement recommendations.

Response: Thanks for pointing this out! We agree it's very important to account for drainage infrastructure, and indeed this point has come up in our conversations with New York City decision-makers. We do so in two ways. First, we use the Department of Environmental Protection (DEP) Predicted Stormwater Flood Maps, which incorporate data on the city's sewer network: in particular, these predictions use 13 different models, one for each hydraulically independent area of the city, and were developed under the assumption of a fully functioning drainage system[3,4]. We use these predictions both to validate our flood detections, and as a covariate in our Bayesian model, as described in Sections 2.1, 2.2, and 2.3.2, thereby incorporating data on the sewer network. We have added these citations, and some of the details above, to the paper, in Supplementary Information §A.2.1.

Second, in response to your concern, we have incorporated data on *catch basins* — specifically, catch basin locations and complaints — into our analysis. As with the other covariates we analyze, we use this data to both (1) validate our flood detections and (2) as an input into our Bayesian model. We find that our flood detections are significantly *negatively* correlated with catch basin density, suggesting that that insufficient stormwater drainage infrastructure density is associated with elevated flood risk, and representing a potential policy intervention point. We also find that our Bayesian model predictions remain largely stable when incorporating the additional data on catch basins, likely because the model already makes use of many other covariates, some of which implicitly already encode information about drainage, as described above. Please see Supp A.2.1 ("Catch Basins" paragraph) for more details on the data, and Supplemental Figure S4 for experimental results.

[3] NYC Department of Environmental Protection. *2024 Stormwater Analysis*. en. Tech. rep. 2024. URL: `https://www.nyc.gov/assets/dep/downloads/pdf/water/stormwater/2024-stormwater-analysis-report.pdf`

[4] NYC Mayor's Office of Resiliency. *New York City Stormwater Resiliency Plan*. Tech. rep. May 2021. URL: `https://www.nyc.gov/assets/orr/pdf/publications/stormwater-resiliency-plan.pdf`

3. The prompt used for VLM classification "Does this image show more than a foot of standing water?" is overly simplistic. Additionally, the alternative prompts compared in Section B.3 remain too simplistic. Testing more multi-step or complex prompts and comparing their performance would help optimize the VLM's classification accuracy.

Response: Thanks for this point — we agree this is a very natural question! To investigate performance using more complex prompts, we sourced four prompts from similar work ([5]) and evaluated their performance using the same procedure we previously used to assess performance: namely, we manually annotated 250 positive classifications, and 250 of their negative classifications. Only one of the four prompts Tsun-Hua Yang et al. "A Novel Multimodal Large Language Model-Based Approach for Urban Flood Detection Using Open-Access Closed Circuit Television in Bandung, Indonesia". en. In: *Water* 17.18 (Sept. 2025) performed comparably to (but somewhat worse than) our original prompt; the three other prompts, which suggested the usage of contextual environmental cues and were quite long, caused Cambrian-1-13B to hallucinate, resulting in far too many positive classifications (i.e., false detections of flooding). Finally, all four more complex prompts, due to their length, were significantly slower to run on our dataset; some would've required over a week to classify all images. For all these reasons, we chose to use our original prompt in our analysis. However, model performance and optimal prompting strategies are a continually evolving frontier, and we view experimenting with additional prompts and prompting strategies as an intriguing direction for future work. We have discussed this point, and included all experimental results, in Methods M.1.1 ("Comparison to alternate prompts") and Supplementary Information B.4 ("Prompt engineering").

4. In the Introduction, the literature review on "visual models in flood monitoring" should be expanded.

Response: We have expanded this section with additional relevant references and discussion.

*5. Lines 140–163 (content related to the dataset) are more appropriate for the **Methods section** rather than the Results. Relocating this content would improve the manuscript's structure.*

[5]Heng Lyu et al. "Assessing Large Multimodal Models for Urban Floodwater Depth Estimation". en. In: *Water Resources Research* 61.4 (Apr. 2025), e2024WR039494; Tsun-Hua Yang et al. "A Novel Multimodal Large Language Model-Based Approach for Urban Flood Detection Using Open-Access Closed Circuit Television in Bandung, Indonesia". en. In: *Water* 17.18 (Sept. 2025); Zhangding Liu, Neda Mohammadi, and John E. Taylor. "FloodVision: Urban Flood Depth Estimation Using Foundation Vision-Language Models and Domain Knowledge Graph". In: *arXiv preprint arXiv:2509.04772* (2025)

Response: Thank you for the suggestion! We have shortened this passage significantly. To avoid confusing the reader, we believe we do need to keep some content on the datasets at the beginning of the Results section (otherwise the reader will not understand the inputs to the model which we subsequently describe).

6. Lines 273–276: To derive the conclusion that "if the VLM predicts an image is flooded, it is xx more likely to be flooded," the correct comparison should be between $p(y = 1|\hat{y} = 1)$ and $p(y = 0|\hat{y} = 1)$. The current comparison between $p(y = 1|\hat{y} = 1)$ and $p(y = 1|\hat{y} = 0)$ is methodologically inaccurate and may mislead readers about the VLM's discriminative power.

Response: Thanks for looking carefully at this! We believe that the correct comparison to back up the claim "if the VLM predicts an image is flooded, it is xx more likely to be flooded", is indeed between $p(y = 1|\hat{y} = 1)$ and $p(y = 1|\hat{y} = 0)$, as performed in the manuscript. This compares the probability an image is flooded given that the VLM estimates it is flooded ($p(y = 1|\hat{y} = 1)$) to the probability an image is flooded given that the VLM estimates it is not ($p(y = 1|\hat{y} = 0)$) as the sentence describes. Additionally, both the metrics reported are very widely used measures of machine learning performance — in particular, $p(y = 1|\hat{y} = 1)$ is the precision or positive predictive value, and $p(y = 1|\hat{y} = 0)$ is the false omission rate, or one minus the negative predictive value — which are reasonable in our setting due, as Reviewer 3 notes, to our highly imbalanced dataset. We have added further discussion of this point in Supplementary Information §B.2 — in particular, our metrics can be precisely estimated even on our unbalanced dataset, while other measures of performance cannot. We thus do not think reporting these metrics is inaccurate or misleading. Additionally, we do not think that comparing $p(y = 1|\hat{y} = 1)$ and $p(y = 0|\hat{y} = 1)$ will provide additional information to the reader, since we already report the first probability, and the second probability is simply one minus the first. We have discussed additional performance metrics, and justified our choice of primary performance metrics, in Results §2.3.1 and Supplementary Information §B.2.

7. Lines 282–290: The three validation datasets (for additional flood events in New York City and San Francisco) report no false negatives ($p(y = 1|\hat{y} = 0)$), whereas the primary dataset (September 29, 2023, New York City flood) includes such errors. The manuscript should: (1) explain why false negatives are absent in the validation datasets; (2) characterize the image features of misclassified samples (e.g., ambiguous water reflections, occluded street views) in the primary dataset to clarify the VLM's limitations.

Response: Thanks for pointing this out — we agree all these points are useful to clarify! The reason the validation datasets have no false negatives is that true positives (i.e., truly flooded

images) are rarer in the validation datasets, because flooding was less pervasive (2 inches[6] of rainfall on December 18, 2023 and 0.9 inches[7] on January 10, 2024 vs. 8.7 inches[8] on September 29, 2023.) The storms affecting the validation datasets were not as severe as the storm affecting our primary dataset, or were collected towards the tail end of the rainfall event (February 2024 California flooding[9]). Consistent with this, we estimate that the fractions of images that are truly flooded, $p(y = 1)$, in the validation datasets are $3 \cdot 10^{-4}$, $8 \cdot 10^{-5}$, and $4 \cdot 10^{-5}$, as opposed to $7 \cdot 10^{-3}$ in our original dataset. We have added an additional section (Supplementary Information B.2, "Generalization across cities and time periods") to discuss these nuances. We have also included additional analysis characterizing the image features of misclassified samples in the primary dataset, as suggested (Supplementary Information §B.2) which we think provides helpful context; thank you for suggesting this!

8. *Lines 298–300: The rationale for using the 25th percentile to define "high BayFlood risk" is unclear. The manuscript should justify this threshold.*

Response: Good point. We chose to set the threshold for BayFlood risk using the 25th percentile of tracts with confirmed flooding for two reasons. First, this threshold captures most of the tracts that likely truly had flooded images (because 75% of tracts with known flooded images were above this threshold). Second, setting an unduly low threshold would increase vulnerability to outliers (as would be a risk if, e.g., we used the 1st percentile of tracts with confirmed flooded images). We have added this reasoning to the manuscript (Methods §M.4). We agree any such threshold is somewhat arbitrary, however, and so we have additionally reproduced Figures 3A-D, the primary result that depends on this threshold, when using a 10% or a 50% threshold instead of a 25% threshold; see Supplemental Figure S4. Results remain similar; in particular, at both alternate thresholds, we identify large numbers of residents living in areas with detected flooding missed by other methods.

9. *Lines 325–328 and Lines 297–300 convey nearly identical content (both defining "high BayFlood risk" tracts).*

[6] Spectrum News Staff New York City. *Live updates: Heavy rain soaks New York City*. en. 2023. URL: `https://ny1.com/nyc/all-boroughs/weather/2023/12/18/new-york-city-rain-live-blog-december-18-2023`

[7] Wikipedia. *January 10–13, 2024 North American storm complex*. en. Jan. 2026. URL: `https://en.wikipedia.org/w/index.php?title=January_10%E2%80%9313,_2024_North_American_storm_complex&oldid=1331413732`

[8] Wikipedia. *September 2023 New York floods*. en. Jan. 2026. URL: `https://en.wikipedia.org/w/index.php?title=September_2023_New_York_floods&oldid=1332708746`

[9] *Potent Storm Drenches California - NASA Science*. en-US. Feb. 2024. URL: `https://science.nasa.gov/earth/earth-observatory/potent-storm-drenches-california-152414/`

This redundancy should be eliminated to streamline the text.

Response: Thank you! We have rewritten this to streamline the text.

10. Lines 345–348 & Lines 395–397 (details on the risk-adjusted logistic regression for 311 reporting biases) belong in the "Methods section" (as they describe analytical procedures).

Response: Thank you — we have moved many of the details here to Methods M.3, including the mathematical specification. We have kept only the intuitive description of what the risk-adjusted regression procedure does: this is essential for clarity of what follows, since it is not a widely-known statistical procedure.

Additionally: (1) the manuscript does not report the statistical significance of regression coefficients;

Response: Thanks, this is a good point — we have clarified in the caption of Figure 3E that the plot depicts 95% confidence intervals for all regression coefficients, with blue indicating statistically significant ($p < 0.05$) positive coefficients and red indicating statistically significant negative coefficients. We have also reported the statistical significance of all regression coefficients in the "Quantifying biases in 311 reports" section.

(2) it is unclear whether the demographic variable was included in the regression "one at a time" (univariate regression) or "simultaneously" (multivariate regression).

Response: Thanks, we agree this is important to clarify. Each demographic variable was included "one at a time" via univariate regression to avoid collinearity concerns, consistent with past work.[10] This is now explicitly mentioned in the Results section, as well as the new Methods M.3 section detailing the risk-adjusted regression specification.

11. Lines 428–432 and Lines 444–446 (in the Discussion) contain redundant descriptions of the study's application in sensor placement. One of these sections should be removed or condensed.

Response: Thanks, we have condensed the latter passage!

12. Line 553–554: The annotation criterion states that an image is "annotated as positive if it showed definite flooding: namely, the street in front of the vehicle was visible and showed significant flooding." This is counterintuitive—

[10] Gabriel Agostini, Emma Pierson, and Nikhil Garg. "A Bayesian spatial model to correct under-reporting in urban crowdsourcing." Proceedings of the AAAI Conference on Artificial Intelligence. Vol. 38. No. 20. 2024.

flooding typically obscures street details, so it is unclear why a "visible street" is used as a criterion for labeling flooding.

> **Response:** Yes, we agree the wording here could be clearer! We meant simply that the image contained a view of the street in front of the vehicle — i.e., the dashcam itself was positioned at an angle suitable for capturing the street — not that the street was uncovered by water. We have revised this wording.

Reviewer 3

This manuscript presents an innovative approach to urban flood detection by leveraging large-scale dashboard camera (dashcam) datasets combined with vision–language models (VLMs). The study introduces BAYFLOOD, a two-stage framework that first applies zero-shot classification to identify flooded street scenes and then uses Bayesian spatial modeling for prediction refinement. The work addresses major limitations in current flood detection methods (311 reports, FloodNet sensors, and modeled stormwater accumulation with elevation data), particularly the issues of poor spatiotemporal granularity and inequitable coverage. Key contributions include demonstrating improved flood detection accuracy in New York City, quantifying demographic biases in 311 reporting systems, and providing actionable recommendations for optimal flood sensor placement. This is a high-quality and technically sound manuscript that makes meaningful methodological contributions toward the utilization of large-scale dashcam imagery for environmental monitoring. The study addresses an important societal challenge in urban resilience and data equity.

> **Response:** Thank you! We are glad you found the approach innovative, actionable, and technically sound.

However, there are several substantial concerns regarding the authors' understanding of hydrologic processes and the robustness of their validation methods. I recommend acceptance after major revisions, addressing the following key points—particularly the concerns on annotation reliability and spatial granularity.

1. As described in lines 551–555, the manual annotation process relied on a single individual labeling 1,000 ground-truth images to ensure "consistency." While consistency across annotators is valuable, the subjective nature of judging "definite flooding" versus "small puddles" raises significant concerns regarding data reliability. In flood modeling, water depth is a critical parameter in defining inundation, and visual ambiguity can easily affect classification outcomes. It is strongly recommended to (a) assess inter-annotator agreement using a subset of images labeled by multiple annotators, or (b) explicitly acknowledge the limitation of using a single annotator and clearly define the operational threshold for what constitutes "flooding." This would enhance transparency and reproducibility.

> **Response:** Thanks for this suggestion — this is a great idea. We agree that measuring inter-

annotator agreement is a standard validation that would strengthen the manuscript. We have now done so and added the results to Methods M.1.1. In brief, we have a second annotator discuss annotation criteria with our first annotator and then re-annotate samples annotated by our first annotator; we then compute Cohen's κ, a standard measure of inter-annotator reliability. We find $\kappa = 0.62$, indicating "substantial agreement" and supporting our use of these annotations in downstream analysis.[11] As additional measures, when our first annotator annotates an image as flooded, the second annotator agrees 98% of the time; when our first annotator annotates an image as *not* flooded, the second annotator agrees 66% of the time.

2. While the paper emphasizes the fine-grained nature of dashcam data compared to sensors or static maps (line 27), the final analysis is aggregated to the Census Tract level, which remains relatively coarse. The claim that 95% of tracts contain at least 50 images also implies that 5% of tracts have fewer samples, raising questions about data representativeness during flood events. Please justify: - Why the Census Tract level was selected instead of more granular units such as Census Blocks or Block Groups.

Response: Good question — we use Census tracts because using finer spatial granularities, like Census block groups or Census blocks, results in too many spatial units which lack sufficient images for reliable analysis. At the Census tract level, we have very good spatial coverage: only 1.3% of New York City tracts have fewer than 20 images on the day of flooding that we analyze. However, the sparse data problem becomes much more severe at finer spatial granularities: 14.8% of Census block groups, and 69.1% of Census blocks, have fewer than 20 images, precluding reliable analysis. Further, while only 0.2% of Census tracts have *no images*, 3.2% of Census block groups and 14.9% of Census blocks had no images. We therefore chose Census tracts to avoid having large portions of the city for which we could not provide reliable inferences.

Justify: - Whether the number of images per tract is sufficient to capture spatial variability in flooding within those tracts.

Response: Thanks, this is also a good question. We agree that if there are too few images per tract, and most of the tract is not flooded, you might fail to detect flooding in some part of the tract. As discussed above, the benefit of fitting a model at the Census tract level is that the large majority of tracts have quite a few images — in particular, 99.4% of tracts have at least 10 images, 98.7% have at least 20, 94.5% have at least 50, and 81.0% have more than 100. To

[11] Richard J. Landis and Gary G. Koch. "The measurement of observer agreement for categorical data." Biometrics (1977): 159-174.

assess whether this is a sufficient number of images to capture spatial variability in flooding, we conducted a power analysis as follows: we simulated a world in which each image in the tract had a probability p of being truly flooded, and computed the probability that our data would contain at least one flooded image for the tract. In other words, we assess whether we have enough images in most tracts that we have a good chance of having at least one image that shows flooding if it is in fact occurring. We have a 90% chance of having a flooded image in 99.7% of Census tracts that are 50% flooded; for 99.5% of tracts that are 25% flooded; and for 98.5% of tracts that are 10% flooded. This demonstrates that we are quite likely to be able to identify flooding in most tracts that are truly significantly flooded.

Response: As an additional corroborating analysis, we confirmed that within Census tracts, the images we have are not all confined to the same area (which would reduce our ability to detect spatial variability). 91.8% of Census tracts had at least one image in every Census block group they contained, demonstrating that in the vast majority of tracts, the images provide good spatial coverage of regions throughout the tract. Cumulatively, these analyses demonstrate that we have good ability to capture spatial variability at the Census tract level. We have added these additional analyses to the manuscript (Section M.2.4) and referenced them in the Results as well.

Justify: - How the Bayesian spatial model compensates for sparse data, particularly in tracts with low image counts, and whether this compensation is validated quantitatively.

Response: Great question. A major benefit of the Bayesian model (relative to using, e.g., just a simple fraction of images that the VLM detects as flooded in each area) is precisely that it can incorporate multiple mechanisms for dealing with sparse data. In particular, the Bayesian model can (1) smooth across spatial areas using an ICAR model, capturing the fact that flooding is spatially correlated and allowing areas with many images to inform inferences for areas with sparse images; (2) incorporate information from flood-relevant external covariates to further improve inference. To your question about quantitative validation, we do demonstrate that these features improve inference, relative to baseline models that do not incorporate them, as described in the subsections "Bayesian modeling improves out-of-sample prediction" and "Comparison of baseline and Bayesian model performance" and Table S4. We also show that the Bayesian model continues to yield stable predictions even when we significantly downsample the dataset — as described in "Model predictions remain stable even with sparser image data" — demonstrating that the model's inferences are stable even in the presence of significantly sparser data than in our empirical setting (though, as expected, stability begins to decline under very high downsampling ratios of 10

or more). Similarly, we show that model predictions remain stable even when there are very few human-annotated images, as described in "Model predictions remain stable even with very few ground-truth annotations". Collectively, these validations provide several lines of empirical evidence that the theoretical benefits of the Bayesian model do suffice in practice to deal with sparse data.

3. *It is unclear what the temporal and spatial coverage of the data is, and the methodology appears to be constrained by its data source. Dashcams inherently provide only street-level perspectives from moving vehicles. I recommend that the authors explicitly discuss two key limitations in the Discussion section: (a) The method cannot capture inundation occurring away from the roadway (e.g., in parks, private properties, or beneath structures such as bridges that are not visible from the street).*

Response: Thanks for raising this. We agree, and have highlighted this in the Discussion as you suggest.

(b) The method is subject to selection bias during severe flood events, as areas that become too dangerous or inaccessible (e.g., due to road closures) are unlikely to be recorded by dashcam-equipped vehicles. This leads to blind spots precisely where flood risks are highest, meaning the risk estimates reflect only observable incidents rather than the full spatial extent of flood occurrence.

Response: Thanks, this is also a good point, and we have highlighted it in the Discussion. We certainly agree that selection bias is a concern — for example, we see fewer images taken near FloodNet depth sensors during the *peak* of flooding as reported by the sensor; this suggests that severe flooding may indeed reduce dashcam coverage. However, we actually do have a surprising level of coverage of severe flooding — for example, we have images from cars stuck in deep floodwater due to flash flooding. Similarly, dashcams within parked vehicles can be useful sensors during these severe flooding events.

In addition, there is no analysis or discussion of how the vision–language model's (VLM) classification performance varies by time of day or lighting conditions. Because flooding can occur at any hour, image degradation at night—caused by low illumination, reflections, or glare—can substantially affect detection accuracy. I recommend evaluating the model's robustness under nighttime conditions. If possible, the authors should include a validation comparing performance between day and night images; otherwise, they should clearly state that the analysis is limited to daytime imagery due to the challenges of low-light environments.

Response: Great point. In preliminary analyses of our data, we concluded that it was much harder to reliably detect flooding at night, as the appearance of floodwater is more muted and hard to discern. We therefore do not include night time images in our analysis. We now

explicitly discuss this point in Results Section 2.1.

4. The authors state that the proposed approach detected flooded areas missed by the existing static predictive map (NYC DEP stormwater map). However, comparing real-world flooding observations from September 29, 2023, with a static map designed for a specific rainfall magnitude (e.g., a 100-year event) and limited to large, contiguous areas ($>$ 0.25 acres) may mislead readers. The two datasets represent fundamentally different temporal and event-based contexts, and this distinction should be clarified.

Response: Thanks — we agree the purpose of these two data sources is different, and have clarified the distinction as you suggest in Supplementary Information §A.2.1, where we discuss the dataset. To clarify, the version of the stormwater map we use does indeed predict hyperlocal flooding, which is visible via the web map[12] for the resource. We agree that using the version of the floodwater risk map for a 100-year flooding event would be misleading; for this reason, we avoid using the versions of the analysis that account for '2050 sea level rise' and '2080 sea level rise', and instead utilize the moderate flooding scenario (2.13in/hr of rainfall) under current sea level conditions.

In addition, the reference for the NYC stormwater flood map appears to be incorrect. The manuscript cites reference [52], which does not contain the underlying data source. The correct source seems to be the NYC Open Data portal:

Response: Thanks, we have fixed this!

Finally, I recommend revising the Introduction or Related Work sections to include recent advancements in high-resolution and physics-based urban flood modeling, which provide more accurate predictive capabilities. Notable studies include Guo et al. (2020), Schubert et al. (2022), Wing et al. (2017), and Noh et al. (2019).

Response: Thanks very much for these suggestions — we have added them to the Introduction!

5. The authors employ specialized metrics for evaluating the VLM (Positive Predictive Value and False Omission Rate). While these choices are reasonable given the highly imbalanced dataset (0.2% positive cases), the manuscript omits several conventional classification metrics commonly reported for completeness—such as F1-score, Recall, and the Critical Success Index (CSI), which are frequently used in urban flooding studies. The

[12]NYC Department of Environmental Protection. *NYC Stormwater Flood Model Viewer - Moderate Flood (2.13in/hr) with Current Sea Levels.* Oct. 2024. URL: `https://experience.arcgis.com/experience/6f5482ced986448a8ba86a0e9bb8ad88/page/Page-1?views=Moderate-2.13-2%2CModerate-2.13-4`

absence of these metrics or a clear rationale for their exclusion limits the interpretability of the reported performance. I recommend that the authors either (a) provide a more comprehensive set of evaluation metrics for the VLM classification (e.g., in Supplemental Table S2), or (b) include a detailed justification explaining why PPV and FOR were considered the most appropriate and sufficient measures for this highly imbalanced, safety-critical detection context (see Bertsch et al., 2022; Lee et al., 2025).

> **Response:** We're glad you agree the metrics we highlight are reasonable given the highly imbalanced nature of the dataset, and have added the citations you mention to further justify them as primary metrics in our analysis. However, we agree that further discussion of other standard metrics — and more motivation for why we focus in particular on positive predictive value and false omission rate — would be very helpful. We have added an extended discussion of all the metrics you mention in Supplementary Information §B.2 (and included a pointer to this discussion in the Results as well) and justified why we focus on our primary metrics. In brief, the reason we do not focus on metrics like recall (and F1 score, which uses recall as an input, along with related metrics like the critical success index) is that these metrics are very difficult to measure precisely on imbalanced datasets such as ours: in particular, when we compute the 95% confidence intervals for several of these metrics, they are so wide that they are essentially uninformative. In contrast, positive predictive value and false omission rate can be much more precisely estimated, which is why we focus on these.

> **Response:** More generally, we have revised the discussion to highlight that practitioners should not assume recall of our method is perfect for several reasons — our images are only on roads, some roads may be inaccessible due to deep flooding, and classifier recall cannot be precisely estimated. While this is an important point, many of the applications we highlight do not depend on perfect recall: for example, our method is still able to identify many locations that have flooding that is missed by other methods.

6. The core comparison in this study involves relating point-based dashcam detections to polygon-based flood maps. However, the authors do not report any metric to evaluate spatial false negatives—that is, areas identified as flooded on the reference map but not captured by dashcam observations. The absence of such a spatial recall assessment limits the evaluation of the method's completeness and reliability. It is recommended that the authors address this limitation in the Discussion section. They should explicitly acknowledge the methodological challenge of comparing point observations to areal data and, if possible, implement a simple spatial performance measure—such as calculating the proportion of flood map polygons (or total inundated area) with no corresponding dashcam detections. This addition would substantially strengthen the credibility and interpretability of the results.

Response: We agree it would be useful to acknowledge the methodological challenge of comparing point observations to areal data and have added this point where we discuss the stormwater flood maps. We also agree that the difficulty of precisely quantifying spatial recall is an important limitation, and have expanded on this point (as mentioned above) in Supplementary Information §B.2 and in the Discussion. (As a point of clarification, we do not think that "areas identified as flooded on the reference map but not captured by dashcam observations" can necessarily be described as "spatial false negatives". We assume by "reference map" you are referring to the DEP stormwater prediction map — however, as you note, this is a static map that may or may not accurately predict flooding in any particular event. We believe the best way to assess false negatives from our detection method is by directly examining the images themselves to get human-labeled ground truth, as we have done. Please let us know, however, if we misunderstood your question — we are happy to do additional analysis here.)

7. Line 59–62: The cited references [17–19] do not appear to substantiate the statement that "these forecasts can be imperfect, particularly as climate change alters historical weather patterns, diminishing their reliability and effectiveness in predicting future flood scenarios." Please revise this section to ensure that the references directly support the claim or provide additional citations that do so.

Response: Thanks for taking a careful look at this! We have chosen references that more precisely support our claims.

8. The manuscript does not clearly explain why the validation for the San Francisco dataset yields a substantially lower Positive Predictive Value (0.143) compared to New York City. This raises concerns about the consistency and generalizability of the proposed method across different geographic contexts, rainfall intensities, and event characteristics. The authors should clarify potential causes of this discrepancy and discuss the implications for the model's robustness and transferability.

Response: Thanks, this is a great point and we agree it could be more clearly explained. There are a couple reasons the estimated PPV in San Francisco is so low. First, true positives (i.e., truly flooded images) are rarer in the validation datasets, because flooding was less pervasive. The storms affecting the validation datasets were not as severe as the storm affecting our primary dataset (2 inches[13] of rainfall on December 18, 2023 and 0.9 inches[14] on January

[13] Spectrum News Staff New York City. *Live updates: Heavy rain soaks New York City*. en. 2023. URL: `https://ny1.com/nyc/all-boroughs/weather/2023/12/18/new-york-city-rain-live-blog-december-18-2023`

[14] Wikipedia. *January 10–13, 2024 North American storm complex*. en. Jan. 2026. URL: `https://en.wikipedia.org/w/index.php?title=January_10%E2%80%9313,_2024_North_American_storm_complex&oldid=1331413732`

10, 2024 vs. 8.7 inches[15] on September 29, 2023.), or were collected towards the tail end of the rainfall event (February 2024 California flooding[16]). Consistent with this, we estimate that the true fraction of flooded images in San Francisco is $4 \cdot 10^{-5}$, as opposed to $7 \cdot 10^{-3}$ in our primary analysis dataset. (One positive impact of the lower prevalence of true positives is that the estimated false omission rate in the validation datasets is also very low.) Second, the PPV in San Francisco is estimated with relatively fewer images (in part because there are far fewer dashcam images in San Francisco than in our primary analysis dataset in New York — 24,000 vs 926,000) leading to noisier estimates. We have added this discussion to the paper (Supplementary Information B.2, "Generalization across cities and time periods"), and specifically noted the challenge of assessing generalization.

We thank the reviewers and editor again for their thoughtful consideration of our manuscript and useful suggestions for improving it. We are very happy to incorporate any further suggestions or respond to any additional questions.

[15] Wikipedia. *September 2023 New York floods*. en. Jan. 2026. URL: `https://en.wikipedia.org/w/index.php?title=September_2023_New_York_floods&oldid=1332708746`

[16] *Potent Storm Drenches California - NASA Science*. en-US. Feb. 2024. URL: `https://science.nasa.gov/earth/earth-observatory/potent-storm-drenches-california-152414/`

Emma Pierson
Assistant Professor
Department of Electrical Engineering and Computer Sciences
Computational Precision Health Program
emmapierson@berkeley.edu
https://people.eecs.berkeley.edu/ emmapierson/

We are grateful to the reviewers and editor for their continued assessment of the manuscript, and are also very glad to see that Reviewers 1 and 2 feel all their suggestions have been addressed! We provide a point-by-point response to Reviewer 3's remaining suggestions and questions below.

The authors incorporated an inter-annotator agreement analysis following my previous suggestion. While the reported Cohen's kappa value (0.61) is interpreted as "substantial agreement," it can also be classified as "moderate" according to McHugh (2012). Given that this dataset serves as benchmark data, a more rigorous standard of agreement should be applied. Furthermore, the agreement rate for the "not flooded" class is relatively low (66%), which is concerning. This may stem from the lack of a clear quantitative threshold or rule to distinguish between "flooded" and "not flooded," suggesting that the benchmark labels may have been assigned subjectively.

Response: Thanks for the careful feedback here. We realized that our previous response reported an incorrectly low measure of interannotator agreement - specifically, it reported the agreement between *the VLM and the first human annotator*, when what we really wanted was the agreement between *the first human and the second human annotator*. We sincerely apologize for this oversight! Fortunately, the agreement between the two human annotators ($\kappa = 0.84$) is much better than the agreement between the human and the VLM ($\kappa = 0.61$, as we reported previously), which makes sense and supports our method's reliance on human annotations as an additional source of ground truth. The value of $\kappa = 0.84$ for human annotators indicates "almost perfect" agreement (according to Landis et al's classification)[1] or "strong" agreement (according to McHugh's classification).[2] Overall, this suggests that these annotations are reliable enough for use.

As additional verifications, we gathered annotations from a third human annotator and again verified that the agreement metric with the first annotator was very high ($\kappa = 0.96$). We also computed another measure of interannotator agreement — Fleiss's κ — which is again very high (0.88). Collectively, these validations demonstrate that interannotator agreement is quite high, supporting our reliance on human labels. Thanks again for pushing us to better explore this point, and we have added these results to the paper (Section M.1.1)

.

[1] Landis and Koch. The measurement of observer agreement for categorical data. *Biometrics*, 1977.

[2] McHugh. Interrater reliability: the kappa statistic. *Biochemia Medica*, 2012.

The authors emphasize the fine-grained nature of dashcam data compared to other approaches; however, their response also indicates that the proposed method does not provide sufficient granularity at the census tract level. This limitation should be more explicitly acknowledged, particularly in terms of spatial coverage. While the data may be fine-grained at the image level, its spatiotemporal coverage appears limited.

Additionally, it would be valuable to evaluate whether image density is associated with socioeconomic characteristics (e.g., fewer images collected in underserved communities), as highlighted by Desouza & Kinney (2021).

> **Response:** Thanks for this suggestion! Nexar's dashcam density is correlated with ridesharing usage, which indeed may correlate with socioeconomic factors. To assess this, we calculated the Pearson correlation (across Census tracts) between between the demographic features in Figure 3E, and the dashcam image density in that Census tract. Although the correlations are statistically significant, they are not that strong: for example, the correlation between log-transformed image density and the fraction of residents who have a Bachelor's degree is weakly positive ($R^2 = 0.11$) as is the correlation with household income ($R^2 = 0.03$). We note that, even though image density varies across tracts, one benefit of our large dataset is that most tracts still have many images; our Bayesian model also mitigates concerns about data sparsity by sharing data across areas. We now report, describe and visualize these correlations (Figure S6) and discuss the socioeconomic skew of the dashcam data in the Discussion.

Given that census tracts can span several square miles, a small number of images may not adequately capture within-tract variability. Although the Bayesian spatial model helps address data sparsity, it remains unclear whether the image density is sufficient to support analysis of urban flooding, which is highly dynamic and rapidly changing.

> **Response:** Thanks, this is a good point. Census tracts can indeed span several square miles in rural areas, but in New York City, tracts span 0.13 square miles on average, much smaller than the nationwide number. We thus believe the concern about tracts spanning several miles is less worrisome in our dataset, which we believe is sufficient to capture spatial variability within New York City. However, we acknowledge that the same number of images would not work for rural areas, motivating our focus on urban areas, and have expanded our acknowledgment of this point in the Discussion.

I agree that the F1 score is not appropriate for imbalanced datasets. However, the Critical Success Index (CSI), which excludes true negatives, provides a more informative measure of performance under class imbalance and should be considered.

Response: Thanks for suggesting this metric! We agree it is useful and did in fact explore it in our earlier round of revisions, in response to your previous comment; we apologize for not mentioning this more explicitly in our last revision. We found that CSI suffers from the same issue we noted in other metrics: it has an extremely wide, uninformative confidence interval (from 0.07 to 0.66) when estimated on our dataset. This wide confidence interval occurs because CSI incorporates false negatives, and the number of false negatives is very difficult to estimate precisely from a random sample on our dataset, because the vast majority of images are classified negative and most, though not all, are true negatives. (Please see Supplementary Information B.2, "Choice of performance metrics", for further details of this point.) Because the confidence interval for CSI is so wide as to be uninformative, we do not rely on it as a measure of performance.

We thank the reviewers and editor again for their thoughtful consideration of our manuscript and useful suggestions for improving it. We are very happy to incorporate any further suggestions or respond to any additional questions.
